# Photophysical, thermal, and DFT studies on a tetraaryl-azadipyrromethene ligand and its zinc(II) complex

**DOI:** 10.55730/1300-0527.3626

**Published:** 2023-10-10

**Authors:** GÖKHAN SEVİNÇ

**Affiliations:** Department of Chemistry, Faculty of Science, Bilecik Şeyh Edebali University, Bilecik, Turkiye

**Keywords:** Tetraaryl-azadipyrromethene zinc(II) complex, density functional theory calculation, singlet oxygen generation, electron-hole analysis, interfragment charge transfer

## Abstract

An azadipyrromethene ligand (**H1**) and homoleptic zinc(II) (**H1-Zn**) complex were synthesized. The resulting structures were elucidated by NMR, FTIR, and HRMS techniques. The photophysical properties and effects of complexing the zinc(II) atom to azadipyrromethene ligands in solution were studied by means of UV-Vis absorption and fluorescence spectroscopy. Experimental findings were elucidated using density functional theory computations and interfragment charge transfer (IFCT) and electron-hole analyses. The fluorescence features were found to be negligible. The ligand molecule decayed at a rate of 3% while the complex decayed at 2% upon photoirradiation based on photostability experiments. The singlet oxygen quantum yields of the ligand and complex were calculated as 0.127 and 0.233, respectively, signifying low photodynamic activity. The charge transfer transitions were determined between reciprocal ligands responsible for the red shift of the main absorption band by IFCT and electron-hole analysis. Compounds in an inert N_2_ atmosphere demonstrated high thermal stability. Although the thermogravimetric analysis (TGA) and derivative thermogravimetry curves of the complexes were similar, zinc(II) coordination and homoleptic complex formation reduced the degradation temperatures. These findings suggest that azadipyrromethene and the Zn(II) class of chromophores have beneficial features for use in the development of novel photo- and thermostable materials that combine charge transfer with low energy in the visible and near infrared regions.

## 1. Introduction

**Figure 1:**
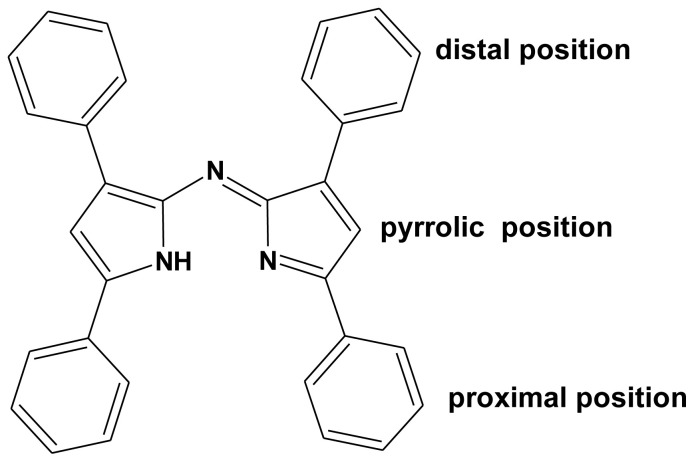
Azadipyrromethene ligand and positions on it.

The purposeful design, synthesis, and photophysical properties of new chromophore compounds are among the areas of interest of the chemical community. For example, optical imaging systems, photodynamic therapy, the design of sensors to be used in the diagnosis of many diseases, and the development of solar cells to be used in energy production are among the leading works. Among the types of dye molecules that can be used in these application areas are azadipyrromethene ligands and their metal/semimetal complexes [1–10]. Azadipyrromethene is an analog formed by replacing the carbon atom in the *meso* position of the dipyrromethene molecule with the nitrogen atom. The main azadipyrromethene ligands usually have four aryl groups in their main skeleton structures. The ligand property is due to the pyrrolic nitrogen atoms in the central region. The part that is close to the center is “proximal” and the part that is farther away is defined as “distal” (Figure 1).

Although its foundations date back to 80 years ago, having been established in the 1940s by Rogers [11], this is a subject that has only recently gained importance and started to be studied more widely. Azadipyrromethenes, which constitute the analog class of dipyrromethenes, have wide absorption bands in the electromagnetic visible spectrum region; their main absorption bands vary between 600 and 900 nm depending on the substituted groups, they have high molar absorption coefficients (>70,000 M^−1^ cm^−1^), and they are used as dyestuffs and are highly soluble in many organic solvents. Since they are open to chemical modifications, their photophysical properties can be varied for different application areas. This ability to change these properties with substituted groups ensures a wide range of applications for these compounds. There are many relevant studies in the literature due to the highly fluorescent properties of azadipyrromethene-BF_2_ complexes, also called aza-BODIPY compounds [12–17]. Although they are overshadowed by semimetal boron complexes, some of the metal complexes of such ligands, excluding boron difluoride and including zinc(II), have been synthesized and their optical properties were determined in a few studies in recent years [6,7]. Accordingly, the effects of metal coordination on both the spectroscopic and photophysical properties of the compounds were elucidated using density functional theory (DFT), X-ray crystal analysis, and absorption spectroscopy.

The transition-metal coordination chemistry of these ligands, which are noted for their intense absorption in the red region of the spectrum, has remained underexplored. Coordination of azadipyrromethenes with Zn(II) gives M(L)_2_-type homoleptic complexes with distorted tetrahedral geometry [18,19]. In the search for beneficial photophysical features, thermal stability, photostability, and singlet oxygen production properties, a new homoleptic azadipyrromethene metal complex was synthesized. For this purpose Zn(II) was selected as the metal center of choice for comparisons because the closed-shell configuration of Zn(II) allows for faster and simpler optimizations. In addition, the presence of distal hydroxymethyl groups of the ligand was determined for the differentiation and comparison of distal positions.

The spectroscopic experimental results were supported by DFT calculations. The descent of the frontier molecular orbital energies was also confirmed by the density of states (DOS) spectra. Besides the variables investigated in the literature, interfragment charge transfer (IFCT) and electron-hole analyses were also applied using theoretical parameters originating from DFT to determine energy transfer dynamics. These calculations provided valuable data for elucidating the photophysical properties of the molecule in the case of singlet excitations for these types of compounds.

## 2. Materials and methods

All reagents and solvents were purchased from Merck Chemical Company (Darmstadt, Germany) and used as received without further purification. Qualitative monitoring of the reactions was performed using thin-layer chromatography (TLC), and for this purpose, TLC silica gel plates (Merck, Kieselgel 60, 0.25 mm thickness) with F_254_ indicator were used and visualized by UV lamp. Melting points were determined on a SMP30 Stuart device (Sigma-Aldrich, St. Louis, MO, USA). Mass spectral analyses of the resulting compounds were performed with an Agilent 6224 LC/MS-High Resolution Mass Time-of-Flight (HRMS) spectrometer without using a separation column (Agilent Technologies, Santa Clara, CA, USA). A PerkinElmer 100 spectrometer equipped with an ATR unit (PerkinElmer, Waltham, MA, USA) was used for the FTIR spectra of the compounds in the range of 650–4000 cm^−1^. ^1^H NMR spectra were obtained with a Bruker Avance 500 MHz spectrometer (Bruker, Billerica, MA, USA) in DMSO-d_6_ with TMS as an internal standard while ^13^C NMR spectra were recorded at 125 MHz in the same solvent. Chemical shifts (δ) were given in ppm relative to solvent peaks (DMSO ^1^H: δ 2.50; ^13^C: δ 39.51). The coupling constants (*J*) were reported in hertz. Thermogravimetric analysis (TGA) and differential thermal analysis (DTA) curves of the compounds were obtained by heating the compounds with an EXSTAR SII TGA/DTA 7200 TG/DTG device (Seiko Instruments Inc., Chiba, Japan) under nitrogen flow with a heating rate of 10 °C/min in the range of 20–1100 °C. The synthesis procedures of compounds **H1** and **H1-Zn** and data on their chemical characterizations are given in the Supporting Information section.

The steady-state UV-Vis spectra of the tetraaryl-azadipyrromethene ligand (**H1**) and zinc complex (**H1-Zn**) were recorded on a Shimadzu UV-1800 scanning spectrophotometer (Shimadzu Corp., Kyoto, Japan). Fluorescence spectra of the studied compounds (5 × 10^−6^ M in THF) were measured with a PerkinElmer LS55 spectrophotometer. Quartz cells of 1 cm were used to perform general absorption and fluorescence measurements at 25 °C. The solvents used in the preparation of the solutions were of analytical grade. Baseline-corrected UV-Vis spectra were collected between 200 and 1100 nm while fluorescence spectra were collected between 200 and 900 nm (excitation and emission slit widths = 10.0 and 8.0 nm, respectively). See the Supporting Information for the fluorescence quantum yield measurements of the compounds.

### 2.1. Photophysical measurements in solution

Singlet oxygen generation experiments were performed for the compounds in CH_2_Cl_2_ with 1,3-diphenylisobenzofuran (DPBF) as a chemical singlet oxygen trap [20–22]. The solutions were irradiated with a white LED light. The degradation of DPBF was monitored by absorption spectra at 414 nm and Φ_Δ_ values were calculated with the following equation:


(1) $$\Phi_{\Delta }=\Phi_{\Delta }\text{ref}\times \frac{\text{k}}{\text{k }(\text{ref})}\times \frac{\text{F}(\text{ref})}{\text{F}}$$

k and k(ref) denote the rate constants of DPBF in the presence of reference methylene blue (Φ_Δ_ = 0.57 in CH_2_Cl_2_) and the sample, respectively. The slope of the line was calculated by the linear fit of the obtained data. F and F(ref) are the absorption correction factors at 600 nm, which is given by F = 1 – 10^−Abs^. Abs signifies the absorbance of the compounds and methylene blue (MB) at 600 nm.

Photostability experiments were performed for compounds **H1** and **H1-Zn** in circular and capped glass tubes with diameters of 1 cm. The sample solutions in acetonitrile were irradiated with a 120-W mercury lamp under room conditions. To eliminate the heat and absorbance of short-wavelength light, a cold NaNO_2_ trap solution (5% w/v, 50 mL) was set between the cells and the lamp. The distance between the light source and the cell was fixed at 5 cm. The irreversible photobleaching of the dyes at the main absorption peak (600 nm and 594 nm for **H1** and **H1-Zn**, respectively) was monitored as a function of time. Acetonitrile solutions of compounds **H1** and **H1-Zn** were deoxygenated with nitrogen gas just before the measurements.

### 2.2. Computational studies

All DFT calculations and visualizations were carried out with Gaussian 09 Rev. C.0 [23,24] and GaussView 5.0.9 [25], respectively. The geometries of the compounds were optimized by employing the hybrid B3LYP functional and mixed basis sets, i.e., the 6-311G(d,p) basis set for the nonmetallic structure of **H1** and the Los Alamos effective core potential basis set (LANL2DZ) for the zinc-including structure of **H1-Zn** in the ground state in the gas phase. TD-DFT computations were used to obtain the vertical excitation energies and oscillator strengths at the optimized ground-state equilibrium geometries at the B3LYP/LANL2DZ theoretical level in the gas phase. Using data from TD-DFT calculations in the gas phase, Multiwfn 3.8 [26] software as a multifunctional wavefunction analyzer was used to calculate the IFCT percentages. Hole-electron analysis of the related compounds was performed with the same software based on the first excited state energies from the TD-DFT calculations. In order to increase the accuracy of the configuration coefficients in TF-DFT calculations, the *IOp(9/40=4)* keyword was used.

DOS diagrams of the investigated compounds were obtained with GaussSum v3.0 software [27].

## 3. Results and discussion

### 3.1. Synthesis and characterization

The −1 and −7 hydroxymethyl-substituted azadipyrromethene ligand (**H1**) and corresponding zinc(II) complex (**H1-Zn**) were synthesized over a sequence of steps as shown in the Scheme. The ligand was synthesized in three steps [8–10]. The aldol condensation of the corresponding 4-(hydroxymethyl)benzaldehyde with acetophenone resulted in the formation of chalcone, which is a *β*-unsaturated carbonyl compound, and then the addition of nucleophilic nitromethane to it by Michael addition reaction followed by a condensation reaction with an ammonium source in refluxing *n*-BuOH afforded the corresponding azadipyrromethene. In the last step, the **H1-Zn** complex was readily obtained by refluxing with ligand with the addition of a stoichiometric amount of zinc salt in butanol [6,10]. TLC analysis showed a new single spot corresponding to the desired compound and the disappearance of the spot corresponding to the starting ligand. Although stepwise reactions are needed, time-consuming processes such as column chromatography are not required as the azadipyrromethene and the final metal complex precipitate in *n*-BuOH at room temperature. The products precipitate out of the reaction mixture and can be isolated with high purity. The synthesis of **H1** and **H1-Zn** resulted in modest reaction yields of 20% and 63%, respectively. The chemical structures of both were confirmed by ^1^H NMR, ^13^C NMR, FTIR, and HRMS techniques (see the Supporting Information). NMR studies of both gave well-resolved spectra in the typical region of 0–10 ppm. In ^1^H NMR, compound **H1** showed a singlet at 7.01 ppm confirming that the structure is symmetrical for the −2 and −6 pyrrole protons; a doublet at 4.62 ppm for the −CH_2_ protons of −CH_2_OH, a doublet at 5.28 ppm for the -OH protons, and four sets of resonances in the region of 7.10–7.88 ppm for protons of the −1, −3, −5, and −7 phenyl groups were compatible with the number of protons in the structure. The ^1^H NMR spectrum of **H1-Zn** is very similar to that of **H1**. Contrary to expectations, no chemical shift was observed in ^1^H NMR. However, upper field shifts were obtained in the ^13^C NMR spectrum of complex **H1-Zn**. In particular, it was observed that the pyrrolic -α carbon atoms (at -α positions on the pyrrole rings) shifted to the upper field in ^13^C NMR, which could be associated with the electron-withdrawing *s* character of the *sp^3^* hybridized Zn(II) atom after complexation. Therefore, the chemical shift observed at 155.5 ppm of the alpha (-α) carbon in the **H1** indacene structure shifted to 160.4 for **H1-Zn**. Similarly, the peak at 143.2 ppm shifted to 145.0 ppm at another proximate carbon atom close to nitrogen. Changes in the retention factor (RF) in TLC, mass, and UV-Vis spectra confirmed the structure. The RF values were 0.57 and 0.48 in MeOH:CHCI_3_ (5%, v/v) for the ligand and complex, respectively. **H1-Zn** migrated up the TLC plate more slowly because of increased hydroxyl groups that bind more strongly to the stationary phase. Thus, the complex compound was clearly separated in the elution solution by column chromatography.

In the HRMS spectra, expected molecular ion peaks were observed at 508.21010 and 1081.33529 (m/z), respectively, in accordance with the oxygen, carbon, and zinc isotopes in the studied structures. It was observed that the M+1 peak intensities increased significantly, especially for compound **H1-Zn** with more carbon atoms and zinc metal. The FTIR spectra are presented in the Supporting Information and include the significant wavenumbers. In the FTIR spectra of the compounds, the peak at 1662 cm^−1^ of the C=O vibrations in the chalcone derivative, which is the first step’s product, was not observed as expected in other ligand and complex structures. Again, strongly associated hydroxyl stretching vibrations were observed in the form of a broad band at 3310 cm^−1^, while the relevant vibrations were observed at 3281 cm^−1^ in the products. C=N vibration peaks at 1544 cm^−1^ were observed as being of weak or medium intensity. The FTIR spectra of compound **H1-Zn** indicated that introduction of the Zn(II) ion to free-base azadipyrromethene did not significantly change the vibration modes. In contrast to ligand **H1**, where the C=N stretching vibration appeared as two signals at 1586 and 1593 cm^−1^, compound **H1-Zn** showed a broad band (1606 cm^−1^) that supported Zn(II) coordination.

### 3.2. Photophysical properties

**Figure 2:**
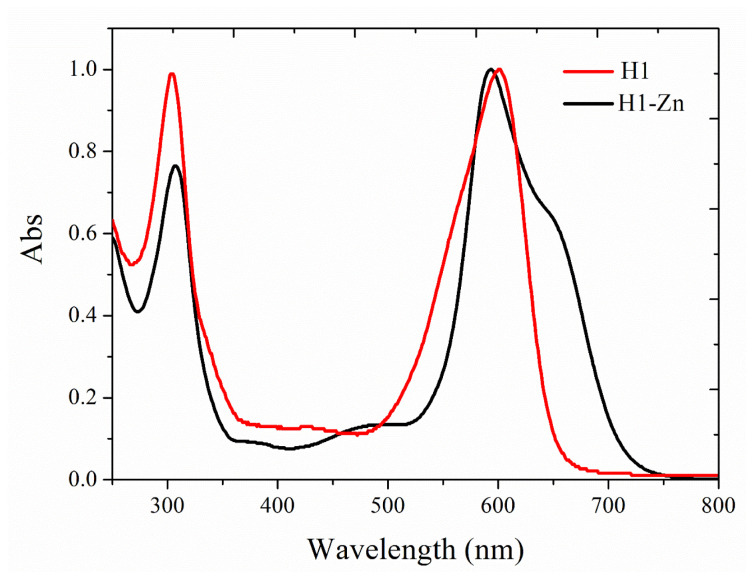
Normalized absorption spectra of **H1** and **H1-Zn** (5 × 10^−6^ M) in THF.

UV-Vis absorption and fluorescence spectroscopies were used to study the photophysical properties of the compounds. Normalized absorption spectra of the compounds are illustrated in Figure 2 and the corresponding photophysical parameters are given in Table 1. The azadipyrromethene ligand (**H1**) showed an intense transition with a maximum absorbance wavelength at 600 nm along with a high molar absorption coefficient. In addition, low absorption in the range of 400–450 nm and strong absorption centered at 300 nm were also observed. Compared to **H1**, a hypsochromic shift was observed on the main absorption band in the absorption spectrum of the complex (**H1-Zn**), along with a shoulder at 647 nm that came to exist towards the right side of the aforementioned peak. Therefore, the active absorbance region was expanded, resulting in an increase of 18 nm in full width at half maximum (FWHM) values. In addition, an approximately twofold increase in molar absorption coefficients was observed due to metal coordination and 1:2 metal-ligand stoichiometry. With the formation of the homoleptic Zn(II) complex, the size of the conjugation system doubled compared to the free ligand. The presence of π-interactions is also expected to increase the rigidity of the complex [7]. When the ligand and complex were compared with their analogs in the literature [6,8–10], there were similarities in absorption wavelengths and molar absorbances. In this respect, it can be stated that the hydroxymethyl groups in the −1 and −7 positions of the indacene structure practically do not alter the photophysical behaviors in terms of absorption profiles. The redshifts and peak broadening of the metal coordination and absorption wavelengths showed a similar trend.

The fluorescence spectra of the compounds were also recorded in THF solvent. In contrast to aza-BODIPYs, Zn(II) dipyrromethene complexes were adapted long ago to be nonfluorescent [5,6,28,29]. Indeed, the fluorescence spectra of the compounds presented low fluorescence peaks. Fluorescein in ethanol (Φ_F_ = 0.79) was used as the fluorescence standard for fluorescence quantum yield calculations. Free ligand **H1** is a very weak emitter with a fluorescence quantum yield of 0.0054 while the Zn(II) complex has partially quenched fluorescence intensity (Φ_F_ = 0.0023). Therefore, the fluorescence quantum yields were found to be negligible. It is known that charge transfer-induced fluorescence is accompanied by a redshift of light absorbance and emissive maximal wavelength or fluorescence quenching [2,30]. Accordingly, when the fluorescence patterns were compared, a red shift of 135 nm was seen, accompanied by a decrease in intensity at the peak maximum of compound **H1-Zn** (Figure S1). Thus, the diminishing fluorescence of the complex may be ascribed to the charge transfer effect, which will be discussed below in the section on theoretical calculations.

**Figure 3:**
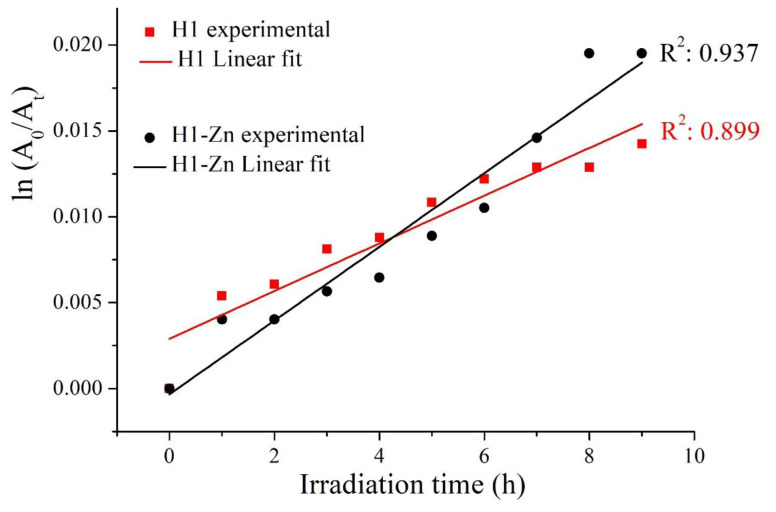
Comparisons of the photodegradation of the dyes in THF.

Photostability is one of the vital properties of chromophores for their uses, especially in solar cell and sensor applications. It is surprising that only a few studies to date have addressed the photodegradation of azadipyrromethene-based dyes, mainly aza-BODIPYs [31–34], and the relationship between photostability and the substituents/coordination at the skeleton of the indacene. Therefore, the photodegradation of the ligand (**H1**) and complex (**H1-Zn**) upon excitation with light was determined by monitoring their maximum absorption bands over irradiation time. The irreversible decreasing of both at the stated absorption peak was obtained as a function of time [35]. For this purpose, the ln(A_0_/A_t_) values were plotted versus irradiation times (Figure 3) in order to obtain the slope value as given in Eq. (2).


(2) $$\text{ln }\frac{{\text{A}}_{0}}{{\text{A}}_{\text{t}}}=\text{k}\text{. t}$$

Here, A_0_ and A_t_ denote the absorbance maximums in the main absorption bands at 600 and 594 nm before and after irradiation for **H1** and **H1-Zn**, respectively. The plots gave a straight line, the slope of which equaled the apparent first-order rate constant k as 2.14 × 10^−3^ mol/h and 1.39 × 10^−3^ mol/h for compounds **H1** and **H1-Zn**, respectively. The ligand molecule decayed at a rate of 3% while the complex decayed by 2% upon irradiation for 8 h. These results show that both compounds were affected at a low rate, but **H1-Zn** is more robust against photodegradation.

**Figure 4:**
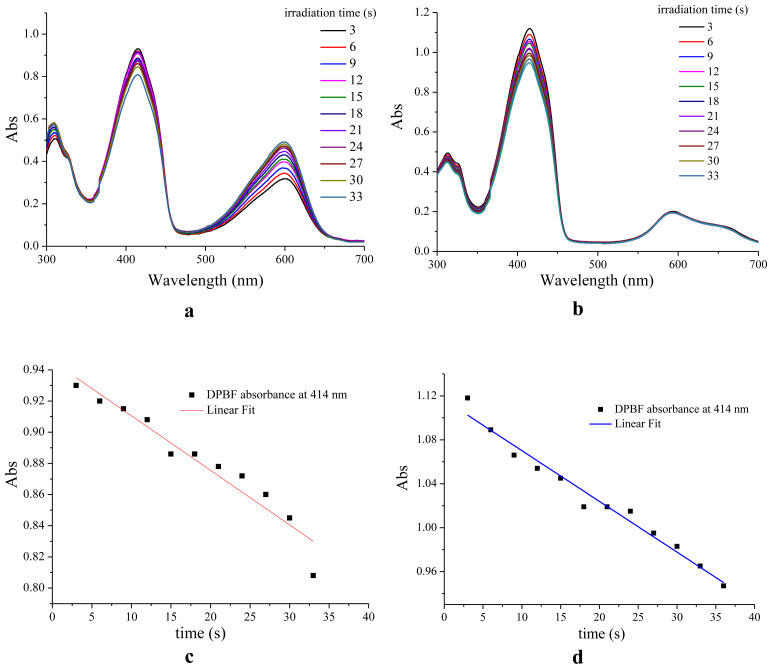
Representative spectra for the determination of the singlet oxygen quantum yields (Φ_Δ_) of **H1** (**a**) and **H1-Zn** (**b**) using DPBF trap, and plots of DPBF absorbance at 414 nm vs. time for **H1** (**c**) and **H1-Zn** (**d**).

Recently, studies on photodynamic therapy applications of new photosensitizer molecules have been increasing [36]. Molecules that do not contain heavy atoms such as bromine are particularly important. Therefore, the singlet oxygen production efficiencies of the compounds were also determined. For this purpose, a comparative method was used [12,21]. The changes of UV-Vis spectra of the chemical singlet oxygen trap (DPBF) in the presence of **H1** and the corresponding Zn(II) complex by light and resulting decays in the main absorbance band of DPBF at 414 nm against time are given in Figures 4a–4d.

As can be clearly seen by comparison with Figures 3a and 3b, the absorbance band of compound **H1** at 600 nm decreased in the presence of singlet oxygen while **H1-Zn** was nearly completely intact. In contrast, the absorption band of DPBF at 414 nm was decreased perceptibly for both. The singlet oxygen quantum yields (Φ_Δ__)_ of compounds **H1** and **H1-Zn** were calculated as 0.127 (12.7%) and 0.233 (23.3%), respectively, when methylene blue (MB) was used as a reference (Φ_Δ_ = 0.57 in DCM) (Table 2). Although the efficiencies of both compounds were low, the metal coordination increased in value approximately two times compared to the ligand. Although they do not contain heavy atoms to allow for spin-orbital coupling resulting in singlet oxygen production, the values obtained here could be examined with triplet formations based on intramolecular charge transfer. The abilities of the compounds to produce singlet oxygen to some degree could be related to the triplet formations. Therefore, DFT calculations were carried out both to determine the photophysical characteristics and to interpret the singlet generation capabilities of the compounds.

### 3.3. Computational studies of the photophysical properties of the compounds

DFT is an effectively used method in computational quantum mechanical modeling to investigate electronic structures of multiparticle systems in both the ground and excited states. In order to reveal the photophysical characteristics of the compounds, DFT and time-dependent DFT (TDDFT) approaches were applied due to their compatible results in the literature for geometry optimizations and excitation analysis of azadipyrromethene-type complexes including aza-BODIPYs and metal complexes [7,37–39]. Initially, the azadipyrromethene skeleton takes a planar geometry based on the optimized geometry. There are low dihedral angles between the phenyl groups at the −1, −3, −5, and −7 positions of the compound and the core structure such that the dihedral angles between the phenyl rings (close to the pyrrole carbons) and the azadipyrromethene core are 15° and 27° for the proximal (−3 and −5) and distal (−1 and −7) positions, respectively. This can be interpreted as the groups attached to the proximal positions due to the smaller angle being more dominant in the photophysics of the azadipyrromethene ligands, especially with better π–π interactions with the core structure. Therefore, the electron distribution in the molecular orbital diagrams of both molecules appears to be spread over the planar core structure and proximal positions. The complex adopts a pseudo-tetrahedral geometry. Moreover, as seen in Table S1 in the Supporting Information, considering the dipole moments of the molecules, it was determined that the dipole moment of the ligand molecule (**H1**) decreased by more than 3 times, which is quite compatible with the homoleptic 2:1 complexation (**H1-Zn**). The change of dipole moment generally brings the alteration of the frontier orbitals of HOMO and LUMO. The frontier molecular orbitals and related dominant transitions are given in Figure 4, which are in good alignment with the experimental results. Theoretical UV-Vis spectra are given in Figure S13.

Accordingly, the HOMO and LUMO orbitals are spread throughout the molecule for **H1**, but they are more dominant in the azadipyrromethene core and the −3 and −5 positions. The presence of hydroxymethyl substituents at distal positions leads to partial expansion of the HOMO-1 and HOMO-2 frontier orbitals, which indicates the low effectiveness of substituents at distal positions in the low-energy absorption region. Although the main transition is H → L, dominant transitions occur as both H → L (62%) and H-1 → L (33%) due to electronic excitation from the singlet energy levels. Electrons due to these transitions are more dominantly localized in the central indacene part of the molecule. The main absorption band observed in the experimental absorption spectrum of the compound belongs to the corresponding transitions. The excited states of the compounds were also studied with TD-DFT calculations (Table S1).

The triplet T_1_ state energy level of **H1** was calculated as 1.15 eV (1075 nm). This transition may theoretically produce singlet oxygen (^1^O_2_), which means that **H1** can be used as a triplet photosensitizer. Indeed, the singlet oxygen quantum yield (Φ_Δ_ = 0.13) was obtained, even though it was lower relative to the reference compound (Φ_Δ_ = 0.57 MB).

**Figure 5:**
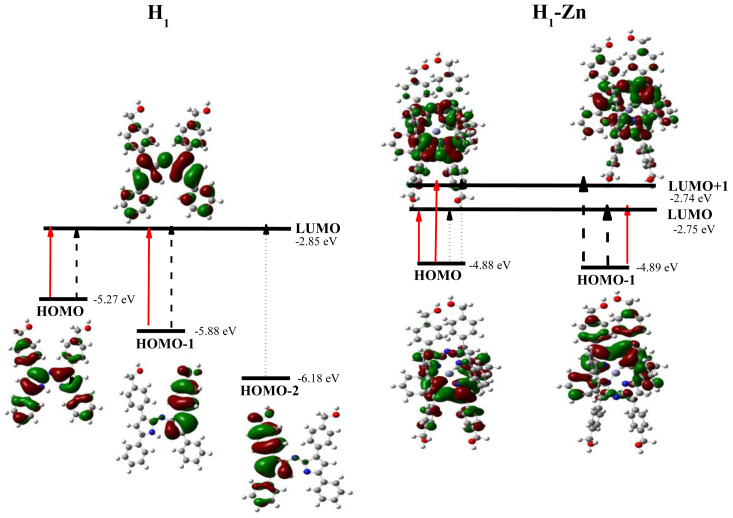
Selected frontier molecular orbitals (isosurface value = 0.02 a.u.) and TDDFT-calculated optical parameters of compounds **H1** and **H1-Zn** for the electronic excitations. Depending on the oscillator strengths (*f*), the main transitions are presented in solid red, the secondary transitions with dashes, and the higher energy transitions with dotted arrows.

Based on the optimized geometry of **H1**, the optimized geometry of the metal complex (**H1-Zn**) was obtained (Figure 5; Table S1). In the homoleptic complex, the HOMO and LUMO orbitals are concentrated on a planar core structure that forms the complex center of the molecule and the −3 and −5 positions connected to it. The optimized structure and molecular electrostatic potential (MEP) are presented in Figure S12. The maximum bond lengths to Zn-N atoms in the azadipyrromethene indacene cores were determined from the optimized structure to be 2.0526 Å and 2.0516 Å, respectively. It was observed that the HOMO and LUMO orbital energies increased in terms of electronic energy levels.

As the HOMO orbital energies rise more, transition energies decrease. In addition, while the main transition is H → L in **H1**, an additional H+1 → L transition was observed in **H1-Zn**. Therefore, a redshift is observed in the theoretical absorption spectrum relative to **H1**. This is why the broadening (FWHM of 80 and 98 nm for compounds **H1** and **H1-Zn**, respectively) and low energy absorption were observed in the experimental UV-Vis spectrum of compound **H1-Zn** (Figure 2). The changes of the frontier orbitals were also confirmed by DOS diagrams, which were drawn by plotting the molecular orbital data of the investigated compounds and are given in Figure S2. The DOS diagrams mainly present the composition of the fragment orbitals contributing to the molecular orbitals [40]. In the DOS spectra, after homoleptic complexation with Zn(II), although there are not significant changes, the frontier molecular orbital energy levels approach each other to some degree. Therefore, the H-1 and L+1 orbitals also become active to perform transitions, which coincides with the experimental UV-Vis spectra.

The S_0_ → T_1_ transition energy for triplet excitation in **H1-Zn** is 0.89 eV (1394 nm) (Table S1). Therefore, singlet oxygen production is not expected. However, it may be that the S_0_ → T_3_ (composition H → L+1) transition observed in the main absorption is responsible in this regard because the energy of the relevant transition may enable singlet oxygen production. The energy of this transition (1.67 eV: 741 nm) appears to be sufficient. This situation can be better understood if it is taken into account that the oscillation strength of the relevant transition is the highest. In other words, the most efficient transition is the S_0_ → S_3_ transition for complex **H1-Zn**.

Depending on the excitation, the possibility of intramolecular charge transfer for the molecule in both the ligand and the metal complex must be evaluated. An effective way to determine the charge transfer in the electron excitation process is by IFCT analysis [41] using the Multiwfn multifunctional wavefunction analyzer [26]. The charge transfer percentage (CT%) and its complementary local excitation percentage (LE%), frequently considered in electron excitation studies between fragments, were identified for compounds **H1** and **H1-Zn** for the S_0_ → S_1_ transition. The results are presented in Table 2.

For compound **H1**, during the S_0_ → S_1_ excitation, fragment 1 donates 0.212 net electrons to fragment 2 and 0.047 net electrons to fragment 3. Therefore, the excitation decreases the electron population of the −1 and −7 distal groups by 0.259 in total during the electron excitation process. If the intrafragment electron redistribution of fragment 2 is taken into account (0.3899), the planar azadipyrromethene structure behaves like a π-linker between fragments 1 and 3. Furthermore, because CT (56%) is larger than LE (44%), the related excitation is dominant in terms of the intramolecular charge transfer character. However, the intramolecular charge transfer (44%) is not to be underestimated.

On the other hand, two fragment definitions were made for **H1-Zn**. Accordingly, fragment 1 donates 0.0025 net electrons to fragment 2. If the IFCT matrix of fragment 1 is taken into account, the contributions of this fragment to the hole and electron are 100% and 99.75%, respectively. On the other hand, these values are 0% and 0.25%, respectively, for fragment 2, which forms the central Zn(II) atom. The intrafragment electron redistribution of fragment 1 is highly dominant (0.9975). Because CT (%) is notably larger than LE (%), the results indicate that there is charge transfer in **H1-Zn** for the S_0_ → S_1_ transition. If the net electron contributions related to the charge transfer of the two compounds are compared, it can be said that this transition is of low intensity for **H1-Zn**, consistent with the oscillator strength. As a result, the lower absorption observed for the main absorption band in the experimental UV-Vis spectrum of **H1-Zn** may belong to the charge transfer transitions between reciprocal ligands.

**Figure 6:**
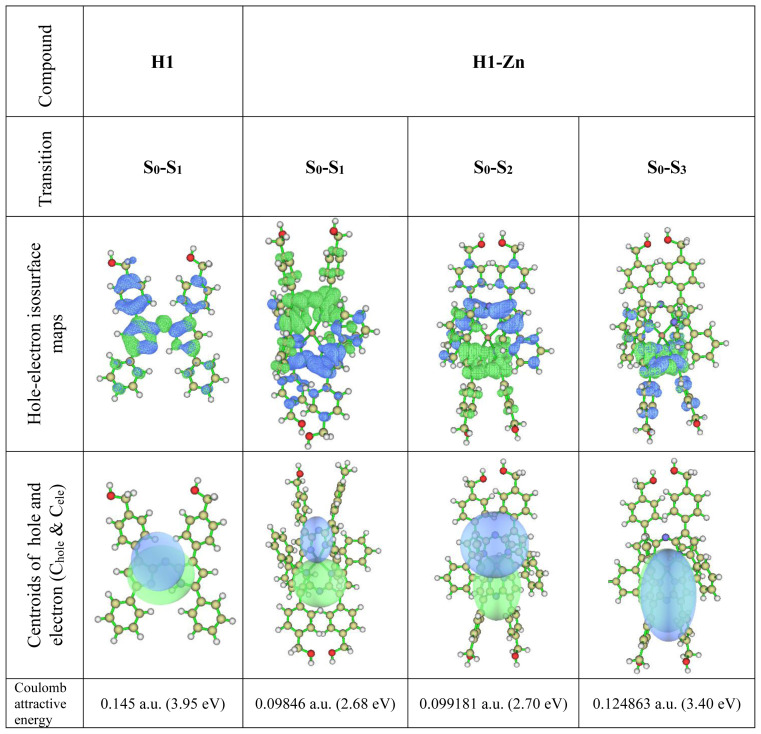
Hole-electron isosurface maps and centroids (C_hole_ and C_ele_) of the holes and electrons of compounds **H1** and **H1-Zn**.

Looking at the intramolecular charge transfers in more detail, electron-hole analysis can be performed using the Multiwfn analyzer. Hole-electron isosurface maps and centroid maps are given in Figure 6 and related data are provided in Table S2. In the figures, green represents the electron distribution and blue represents the hole distribution. Both the hole and electron isosurfaces appear in the pyrrolic indacene core, so there is no doubt that S_0_-S_1_ includes the LE for compound **H1**. However, at the −1 and −7 positions, there are hole distributions that describe the charge transfer between the phenyl groups and indacene core. This result is in good agreement with the IFCT analysis findings presented above. Electron and hole distributions are on the plane along the aromatic pyrrole and phenyl groups. Therefore, it can be inferred that S_0_-S_1_ excitation has a π-π* feature. Meanwhile, it can be stated that nonbonding electron pairs belonging to the oxygen atom contribute to the hole distribution partially while the central metal atom does not have a contribution to either. This may be due to the closed shell configuration of the Zn(II) and the rigid π-conjugate system extended along the two azadipyrromethene ligands via interligand π–π interactions, as noted earlier [6,7,18].

For the **H1-Zn** complex, the data of three singlet transitions were obtained. First of all, it can be stated that the transition between S_0_ and S_3_ is dominant because its f-factor is the highest. The related oscillator strengths are 0.0004, 0.0013, and 0.5534 for the S_0_ → S_1_, S_0_ → S_2_ and S_0_ → S_3_ transitions, respectively (see Table S1). Thus, it can be seen from the data that the HOMO orbital is dominant for the hole. It contributes as much as 80.9%, while the electron is mainly composed of the LUMO orbital, with a contribution of 64.0%. In addition, the H-1 orbital also contributes to the hole partially, while L+1 contributes 35.0% for the electron. This means that the hole contributions of the excited electrons are mostly due to HOMO electrons. In order to visualize the spread of the holes and electrons, isosurface maps are shown in Figure 6. Accordingly, although the distal positions contribute to the hole in the S_0_ → S_3_ transition (the main transition), they almost completely overlap. The main transition in the experimental excitation spectrum belongs to it such that S_0_ → S_3_ is LE excitation with the π-π* feature. This is in agreement with the H → L transition data for singlet excitation given in Table S1. On the other hand, there are differences in the other two low-energy transitions (S_0_ → S_1_ and S_0_ → S_2_). Although their oscillation strengths are low, they are similar in terms of electron/hole contributions. LUMO orbitals are dominant in the electrons and HOMO-type orbitals in the holes. The main dissociation is observed in electron/hole distributions compared to S_0_ → S_3_. As seen in the C_electron_/C_hole_ isosurfaces (Figure 6), the electron/hole pairs are separated. This shows that the relevant transitions have substantial charge transfer characters (CTs), as also indicated in the IFCT analysis. This is also consistent with the low-energy charge transfer transitions seen in the azadipyrromethene zinc(II) complexes with the conjugation of such compounds. Due to the formation of homoleptic Zn(II) complexes, the size of the conjugation system doubles compared to the free ligand [2,6,7]. Coulomb attractive energies (exciton binding energies) between the hole and electron surfaces of the compounds are also in agreement with the redshift of the peak positions corresponding to the transitions of absorption spectra. Therefore, it was concluded that the observation of another absorption band as a shoulder in the low-energy region of the electronic spectrum of compound **H1-Zn** was due to mutual electron transitions (charge transfers) between the azadipyrromethene ligands because d-d transitions are not possible. Hence, the electronic spectrum of **H1-Zn** did not give further absorption originating from the Zn(II).

### 3.4. Thermal properties

**Figure 7:**
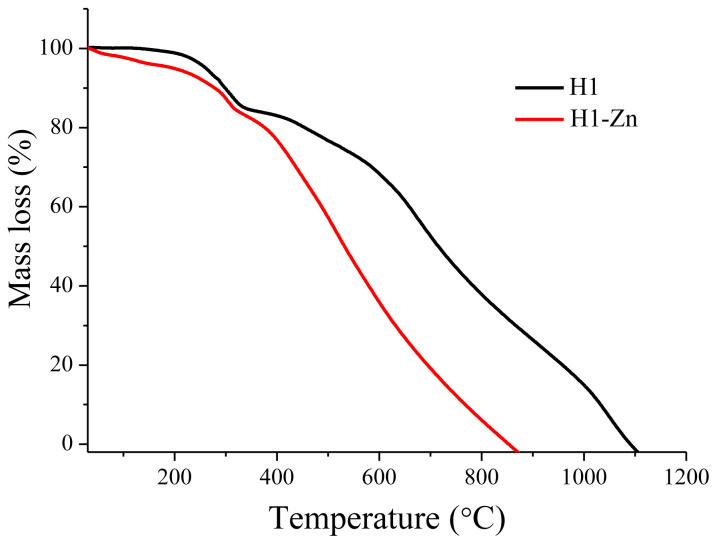
TGA thermogram of **H1** and **H1-Zn** at a temperature ramp of 10 °C/min under N_2_.

The thermal behaviors of the compounds were measured using TGA and DTA. With the TGA and DTA methods, the weight change and heat flux of the materials are simultaneously measured as a function of temperature or time, respectively [42]. Thermal behaviors and decomposition rates of the compounds are given in Figure 7 and Table 3. The compounds demonstrated high thermal stability in an inert N_2_ atmosphere. As can be seen from Table 3, the synthesized compounds show three-step degradation behaviors in the range of 200–1100 °C. The temperature at which the destruction of the compounds begins (T_max_1) declines from 307 to 297 °C for **H1** and **H1-Zn**, respectively. In terms of degradation rates, compound **H1** has a degradation rate between 1.66 and 1.91 (%/min) in the respective degradation steps, while these values are between 1.12 and 2.28 (%/min) for **H1-Zn**. The degradation rate of compound **H1-Zn** increased significantly, especially at 514 °C. The greatest mass loss is observed in this decomposition step and the sample decreases to about 50% of the initial amount. The thermal stability of the compounds was defined by a range of temperatures. Accordingly, when T10, T30, and T50 values were taken into account, it was determined that the thermal stability of the complex compound decreased significantly. While the decrease in thermal stability was 7% at 10% decomposition temperatures, it was calculated as 26% at the T30 and T50 temperatures. **H1** was completely charred at 1090 °C and **H1-Zn** at 851 °C. Remaining char yields were 48% and 81% at 700 °C for compounds **H1** and **H1-Zn**, respectively. Although the TGA and DTG curves of the complexes have similar shapes (Figure 7), Zn(II) coordination and homoleptic complex formation reduce the degradation temperatures, which is not surprising for a macrostructure with a molar mass of 1081.3 g/mol. Additionally, the lower thermal stability of **H1-Zn** in comparison to **H1** may be due to the reduced oxidation potential of the azadipyrromethene core, which initiates the processes of intramolecular oxidation at lower temperatures.

## 4. Conclusion

In this study, an azadipyrromethene ligand was successfully synthesized in three steps starting from an aldehyde derivative and acetophenone. Subsequent treatment with Zn(II) provided the homoleptic complex in moderate yield. When evaluated in terms of substituents, the presence of the hydroxy methyl group, which is a primary alcohol derivative, in the structure may allow some additional reactions on the molecule. For example, by substitution reactions, the binding of halogen atoms to the structure is possible, from which Wittig reactions can take place. In addition, alcohol groups can be oxidized to aldehyde and/or acid groups with temperate oxidizing species. In this respect, hydroxymethyl groups should be considered as active groups in purposeful synthesis designs. The compounds were identified by several spectroscopic techniques that confirmed the predicted structures. Although no practical difference was observed in the ^1^H NMR spectra of the metal complex and the ligand, the HRMS data and ^13^C NMR clearly confirmed the structures. The photophysical properties of the compounds revealed the bathochromic shift of the main absorption band originating from the charge transfer between the ligands. In this respect, the Zn(II) center does not participate in charge transfer processes; it holds the two azadipyrromethene ligands together. Its function is to increase planarity by restricting the rotation of chelate ligands. As a result of DFT calculations, the energy level gaps of the frontier HOMO-LUMO orbitals do not exhibit noticeable changes in altering the maximal wavelengths of light absorption, which is in agreement with the experimental UV-Vis spectra. Nevertheless, it was determined that the electron-hole pairs formed on the cross ligands due to singlet excitations caused higher molar absorption coefficients in the absorption spectrum of the complex and the main absorption band became broader. This shows that the relevant transitions have charge transfer features, as also indicated in the IFCT and electron-hole analyses. The ^1^O_2_ formation abilities of the compounds were also determined by a comparative method with a singlet oxygen trap molecule. It was determined that the compounds have the ability to produce singlet oxygen at a low rate, whereas the homoleptic structure and zinc coordination increase the singlet oxygen production by approximately twofold. Although both compounds were determined to be stable against photodegradation, the azadipyrromethene ligand offered higher photostability. Additionally, it was determined that compounds are very stable in terms of heat and have three-stage decomposition behavior in the range of 200–1100 °C based on TGA measurements in an inert N_2_ atmosphere. Remaining char yields were 48% and 81% at 700 °C for compounds **H1** and **H1-Zn**, respectively. In conclusion, transition metal chelation presents a novel means of tuning the absorption properties of azadipyrromethenes, an emerging class of coordination chemistry with many potential applications both in vivo and in vitro.

## Supporting information

### 1. Synthesis of the compounds

#### 1.1. Synthesis of 3-[4-(hydroxymethyl)phenyl]-1-phenylprop-2-en-1-one (chalcone)

4-Hydroxymethylbenzaldehyde (2.86 g, 21.0 mmol) was dissolved in methanol (100 mL). Acetophenone (2.50 mL, 21.4 mmol) and freshly prepared 2 M methanolic KOH solution (2 mL) were added to the reaction. The mixture was stirred at room temperature for 24 h. The reaction mixture was cooled on an ice bath and neutralized with 1 M hydrochloric acid (4 mL). The solid formed was filtered and washed with 50 mL of distilled water. The final solid was air-dried. Pale yellow solid, yield: 3.25 g (65%), mp: 97–99 °C. FTIR (ATR, cm^−1^) *ν*_max_: 3310 (O-H stretching), 3049 (aromatic C-H), 2913 (aliphatic C-H), 2856, 1662 (C=O), 1607 (C=C aromatic), 1565, 1511 (C=C alkenyl), 1446, 1414, 1334 (C-O), 1305 (O-H bending), 1218, 1116, 1047, 1032, 1019, 982, 818, 774, 754, 689, 654.

#### 1.2. Synthesis of [3-(4-hydroxymethylphenyl)-5-phenyl)-1H-pyrrole-2-yl] [3-(4-hydroxymethyl phenyl)-5-phenylpyrrole-2-ylidene]amine (H1)

To a solution of 2.50 g (10.5 mmol) 3-[4-(hydroxymethyl)phenyl]-1-phenylprop-2-en-1-one (chalcone) in 100 mL of EtOH, 5.43 mL of diethylamine (52.5 mmol) and 2.81 mL of nitromethane (52.5 mmol) were added. The mixture was stirred at 80 °C for 24 h. After this time, excess diethylamine and EtOH were removed in a rotary evaporator. The resulting light-colored oily product was dissolved in EtOH, and water (20 mL) was added and precipitated in the cold. The solid formed was filtered and dried in a vacuum oven at room temperature. This intermediate product was then dissolved in 100 mL of *n*-BuOH. Ammonium acetate (24.2 g, 320 mmol) was added. The mixture was stirred at 120 °C for 24 h. After this time, the mixture was cooled to room temperature and the formed solid was filtered. The precipitate was purified by washing with cold EtOH. Blue solid, yield: 0.54 g (20%), mp: 279–281 °C. FTIR (ATR, cm^−1^) *ν*_max_: 3281 (O-H stretching), 2861 (aliphatic C-H), 1544 (C=N), 1497, 1465, 1445, 1418, 1354 (O-H bending), 1240, 1173, 1134 (C-N), 1005, 958, 902, 808, 761, 679. ^1^H NMR (500 MHz, DMSO-d_6_): d [ppm]: 4.62 (d, *J*: 6.0 Hz, 4H), 5.28 (t, *J*: 6.0 Hz, 2H), 7.01 (s, 2H), 7.11–7.10 (m, 6H), 7.41 (d, *J*: 8.0 Hz, 4H), 7.55–7.53 (m, 4H), 7.88 (d, *J*: 8.0 Hz, 4H). ^13^C NMR (125 MHz, DMSO-d_6_) d: 155.5, 149.5, 143.2, 142.0, 132.1, 131.8, 131.0, 129.9, 128.8, 127.1, 126.9, 116.1, 63.2. HRMS (Q-TOF-ESI) (m/z Calcd: 509.21034 (C_34_H_27_N_3_O_2_), found: 508.20101 [M-H]^−^, Δ = 2.95 ppm).

#### 1.3. Synthesis of bis[3-(4-hydroxymethylphenyl)-5-phenyl)-1H-pyrrole-2-yl] [3-(4-hydroxymethyl phenyl)-5-phenylpyrrole-2-ylidene]amine]zinc(II) complex (H1-Zn)

Compound **H1** (250 mg, 420 μmol) and Zn(OAc)_2_.2H_2_O (44 mg, 200 μmol) were added to 10 mL of n-BuOH and stirred at 120 °C for 3 h. After this time, the reaction mixture was cooled to room temperature and filtered. The solid was washed with 10 mL of cold EtOH and then air-dried. Blue-brown solid, yield: 140 mg (63%), mp: >350 °C (decomp.). FTIR (ATR, cm^−1^) *ν*_max_: 3281 (O-H stretching), 2861 (aliphatic C-H), 1544 (C=N), 1497, 1465, 1445, 1418, 1354 (O-H bending), 1240, 1173, 1134 (C-N), 1005, 958, 902, 808, 761, 679. ^1^H NMR (500 MHz, DMSO-d_6_): d [ppm]: 4.62 (d, *J*: 6.0 Hz, 8H), 5.29 (t, *J*: 6.0 Hz, 4H), 7.00 (s, 4H), 7.11–7.10 (m, 12H), 7.41 (d, *J*: 8.5 Hz, 8H), 7.55–7.53 (dd, *J*: 7.5/2.0 Hz, 4H), 7.88 (d, *J*: 8.0 Hz, 8H). ^13^C NMR (125 MHz, DMSO-d_6_) d: 160.4, 148.0, 145.0, 142.8, 133.1, 132.8, 129.7, 129.6, 128.5, 126.9, 126.8, 117.9, 63.3. HRMS (Q-TOF-ESI) (m/z Calcd: 1080.33417 (C_68_H_52_N_6_O_4_Zn), found: 1081.33524 [M+H]^+^, Δ = 6.25 ppm).

### 2. Fluorescence quantum yield measurements of H1 and H1-Zn

Fluorescence spectra of the studied compounds (5 × 10^−6^ M in THF) were measured with a PerkinElmer LS55 spectrophotometer. Quartz cells of 1 cm were used to perform general absorption and fluorescence measurements at 25 °C. The solvents used in the preparation of the solutions were of analytical grade. Fluorescence spectra were collected between 200 and 900 nm (excitation and emission slit widths = 10.0 and 8.0 nm, respectively). See supplementary material for the fluorescence quantum yield measurements of the compounds. The following equation was used to calculate the fluorescence quantum yield of the compounds:


(1) $$\Phi_{F}=\Phi_{F}(Std)\frac{\text{F}.{\text{A}}_{\text{Std}}.n^{2}}{F_{Std}.A.n_{Std}^{2}}$$

Here, Φ_F_ (Std) is the fluorescence quantum yield of rhodamine B. F and F_Std_ denote the areas under the fluorescence emission curves of the samples and the standard, respectively. A and A_Std_ are the respective absorbances of the samples and standard compound at the excitation wavelengths. n^2^ and n^2^_std_ are the refractive indices of the solvents used for the sample and standard, respectively. Fluorescein in ethanol (Φ_f_ = 0.79) was used as the fluorescence standard for fluorescence quantum yield calculations. In the fluorescence quantum yield determinations, correction for the solvent refractive index (η) was applied [in THF: η = 1.4072]. Fluorescence quantum yields were found to be negligible values that included considerably scattered peaks.

Figure S1 Fluorescence profiles of the compounds in THF.

**Table S1:** Dipole moments (μ), electronic excitation energies (E_v_), corresponding oscillator strengths (*f*), and the main configurations of the low-lying electronic excited states of compounds **H1** and **H1-Zn**.

	Dipole moment (μ/Debye)	Electronic transition	Exciatation Energy (eV/nm)	*f* a	Composition
**H1**	3.4243	S _0_®S _1_	2.28/544	0.4870	H®L H-1®L
		S _0_®S _2_	2.56/484	0.3126	H-1®L H®L
		S _0_®S _3_	2.86/433	0.1463	H-2®L
		T _0_®T _1_	0.712/1742	0.0161	H®L
		T _0_®T _2_	1.3504/918	0.0066	H-4®L
		T _0_®T _3_	1.3972/887	0.0484	H-1®L
		S _0_ → T _1_	1.1530/1075	0.0000 ^c^	H®L
		S _0_ → T _2_	1.9078/650	0.0000 ^c^	H-1®L
		S _0_ → T _3_	2.4635/503	0.0000 ^c^	H-2®L
**H1-Zn**	1.0483	S _0_®S _1_	1.676/739	0.0004	H®L H®L+1
		S _0_®S _2_	1.680/738	0.0013	H-1®L H-1®L+1
		S _0_®S _3_	2.080/595	0.5534	H®L H®L+1 H-1®L
		T _0_®T _1_	0.6434/1927	0.0001	H®L (ß)
		T _0_®T _2_	0.6573/1886	0.0001	H®L (a)
		T _0_®T _3_	0.9041/1371	0.0000 ^c^	H®L+1 (ß)
		S _0_ → T _1_	0.8894/1394	0.0000 ^c^	H®L
		S _0_ → T _2_	0.9029/1373	0.0000 ^c^	H-1®L+1
		S _0_ → T _3_	1.6734/741	0.0000 ^c^	H®L+1

a Oscillator strength.

b Coefficients of the wave function for each excitation. H stands for HOMO and L stands for LUMO.

c No spin-orbital coupling effect was considered; thus, the *f* values are zero.

**Table S2:** Quantitative contributions of holes and electrons of compounds **H1** and **H1-Zn**.

	Transition	MO	Occ	Hole (%)	*Electron (%)*

**H1**	S _0_-S _1_	H-2	2	1.418	0.000
		H-1	2	22.048	0.000
		H	2	75.814	0.000
		L	0	0.000	99.321

**H1-Zn**	S _0_-S _1_	H-1	2	10.202	0.000
		H	2	89.561	0.000
		L	0	0.000	34.181
		L+1	0	0.000	65.758

	S _0_-S _2_	H-1	2	89.080	0.000
		H	2	10.682	0.000
		L	0	0.000	68.407
		L+1	0	0.000	31.533

	S _0_-S _3_	H-5	2	1.446	0.000
		H-4	2	3.455	0.000
		H-1	2	12.749	0.000
		H	2	80.915	0.000
		L	0	0.000	64.031
		L+1	0	0.000	35.006

MO: Molecular orbital; Occ: corresponding occupation number; H: HOMO; L: LUMO.

**Table S3:** Cartesian coordinates for DFT-optimized structure of **H1**.

C	2.0693	−2.58809	−0.07908
C	0.38361	−1.09834	−0.05608
C	−0.19072	−2.36458	−0.08543
H	0.74103	−4.35215	−0.12254
C	2.14212	2.67519	0.04385
C	0.93623	3.40481	0.04565
C	−0.13623	2.50143	0.01564
H	0.85267	4.47136	0.0665
C	0.8501	−3.288	−0.09967
C	0.40262	1.2156	−0.00478
C	3.55687	3.28297	0.07191
C	4.67923	2.44369	0.06802
C	3.7224	4.67419	0.10152
C	5.96713	2.99546	0.09345
H	4.55275	1.38142	0.04558
C	5.01051	5.22609	0.12691
H	2.86546	5.31498	0.10467
C	6.13281	4.38665	0.12287
H	6.82392	2.35453	0.09041
H	5.1371	6.28833	0.14944
H	7.11631	4.80798	0.1422
C	−1.49847	2.83195	0.00778
C	−2.46628	1.81873	−0.02345
C	−1.89284	4.17674	0.0312
C	−3.82815	2.15049	−0.03211
H	−2.16556	0.79201	−0.0407
C	−3.25442	4.50835	0.02275
H	−1.15427	4.95045	0.05555
C	−4.22206	3.49531	−0.00904
H	−4.56714	1.37707	−0.05633
H	−3.55508	5.5351	0.04059
C	−1.56063	−2.65829	−0.09838
C	−2.50156	−1.61998	−0.08201
C	−1.98897	−3.99205	−0.12785
C	−3.87137	−1.91598	−0.09412
H	−2.17415	−0.60158	−0.06028
C	−3.3583	−4.28814	−0.13975
H	−1.27002	−4.78453	−0.14125
C	−4.29958	−3.25029	−0.1228
H	−4.59021	−1.1234	−0.08151
C	3.46042	−3.24832	−0.08683
C	4.61397	−2.45292	−0.065
C	3.57259	−4.64494	−0.1157
C	5.87977	−3.05426	−0.07251
H	4.5285	−1.38656	−0.04273
C	4.83829	−5.24619	−0.12316
H	2.69172	−5.2522	−0.13214
C	5.99186	−4.4508	−0.10159
H	6.76067	−2.44718	−0.056
H	4.92396	−6.3125	−0.14527
H	6.95816	−4.90988	−0.10734
N	1.84828	−1.25618	−0.05175
N	1.85946	1.36403	0.01397
N	−0.27789	0.06431	−0.03548
C	−5.71836	3.85979	−0.01855
H	−5.86413	4.78249	0.50324
H	−6.05337	3.96485	−1.02931
H	−3.6852	−5.30674	−0.16185
C	−5.80433	−3.57676	−0.13566
H	−6.15693	−3.67246	0.87
H	−6.33623	−2.78885	−0.62668
O	−6.01938	−4.80536	−0.83516
H	−6.95741	−5.0094	−0.84267
O	−6.46518	2.82452	0.62609
H	−7.39797	3.05148	0.62034
H	2.52623	0.61896	0.00587

**Table S4:** Cartesian coordinates for DFT-optimized structure of **H1-Zn**.

C	−3.00248	2.05574	2.45765
C	−2.19998	3.21446	2.68227
C	−4.31178	3.87695	2.42797
C	−4.32155	2.50459	2.28572
C	−0.09181	4.56952	2.74456
C	1.29638	4.75425	2.57651
C	1.52198	6.13898	2.45891
C	0.27893	6.76918	2.56126
H	−5.17765	1.89877	2.08066
H	2.46696	6.62107	2.31787
N	−3.06764	4.23603	2.69347
N	−0.80411	3.34432	2.83063
N	−0.62386	5.81757	2.76938
C	−2.66973	0.69914	2.3886
C	−1.36594	0.21378	2.54211
C	−3.72665	−0.18797	2.14489
C	−1.12974	−1.17233	2.44965
H	−0.5577	0.8887	2.72978
C	−3.49486	−1.55752	2.05109
H	−4.72092	0.19187	2.03038
C	−2.19965	−2.05437	2.1916
H	−0.13963	−1.55593	2.57517
H	−4.30929	−2.22698	1.87014
C	2.28965	3.77176	2.51303
C	3.61432	4.17313	2.3191
C	1.97526	2.41138	2.63263
C	4.62526	3.21791	2.19353
H	3.85057	5.21313	2.26241
C	2.99128	1.44934	2.50812
H	0.96499	2.10617	2.81426
C	4.31324	1.85538	2.26045
H	5.63732	3.52833	2.0426
H	2.75699	0.40787	2.59867
C	0.01373	8.14494	2.47139
C	−1.31016	8.60413	2.54788
C	1.06249	9.05954	2.30531
C	−1.58422	9.97641	2.45591
H	−2.11211	7.90614	2.67576
C	0.78772	10.42946	2.21326
H	2.07281	8.71255	2.24836
C	−0.5353	10.88692	2.28822
H	−2.59408	10.32738	2.51186
H	1.58889	11.12792	2.08433
H	−0.74267	11.93327	2.22162
C	−5.39642	4.76209	2.33855
C	−6.6982	4.28595	2.13606
C	−5.16733	6.14114	2.46706
C	−7.77043	5.18592	2.07164
H	−6.87264	3.23498	2.03433
C	−6.2411	7.04025	2.40289
H	−4.17174	6.50671	2.61544
C	−7.54249	6.56125	2.20514
H	−8.76497	4.8219	1.92178
H	−6.06802	8.09134	2.50476
H	−8.36206	7.24889	2.15574
C	5.43127	0.81696	2.05681
H	5.0206	−0.06414	1.61154
H	6.18072	1.2281	1.41093
C	−1.96682	−3.57166	2.05187
H	−2.70602	−3.98266	1.39057
H	−0.99322	−3.75061	1.65305
O	−2.07904	−4.19757	3.33325
H	−1.94259	−5.14289	3.23994
O	6.02032	0.48233	3.31572
H	6.71925	−0.16337	3.17852
Zn	−2.48096	5.97567	3.06909
N	−3.33204	7.08988	1.82373
N	−3.14268	6.65554	4.70438
C	−3.90116	8.23417	2.21832
C	−3.43643	6.86864	0.52741
C	−3.7331	7.87899	4.68506
C	−3.13126	6.11439	5.923
C	−4.39434	8.83254	1.02474
N	−3.99093	8.6757	3.54733
C	−4.10125	7.94822	−0.0276
C	−2.92541	5.73788	−0.12399
C	−4.07008	8.16442	6.02515
C	−3.69098	7.05267	6.79529
C	−2.63257	4.84802	6.26944
C	−5.06296	10.04398	0.82825
H	−4.34134	8.0822	−1.06087
C	−3.73034	4.62175	−0.40278
C	−1.6272	5.79778	−0.64938
C	−4.68321	9.32128	6.52169
H	−3.80991	6.94024	7.8518
C	−2.12531	3.99252	5.27834
C	−2.63177	4.43954	7.61076
C	−4.34603	11.19835	0.47925
C	−6.46387	10.06045	0.77605
C	−3.19854	3.53736	−1.12118
C	−7.14324	11.2115	0.35921
H	−7.01826	9.187	1.04973
C	−1.87762	3.58196	−1.59057
H	−3.80081	2.67645	−1.31062
H	−0.09264	4.75975	−1.72943
C	−5.59443	10.56715	8.39214
H	−4.65547	8.64683	8.55985
C	−5.68548	11.49345	6.1493
H	−4.82195	10.27447	4.6088
C	−1.58944	2.34473	6.97806
H	−1.22087	2.08242	4.87848
H	−2.08943	2.88933	8.99449
C	−6.4226	12.34943	−0.01784
H	−4.47437	13.23076	−0.1993
H	−8.21358	11.21758	0.32462
H	−1.46895	2.7491	−2.1255
C	−5.97098	11.5955	7.51667
H	−5.8032	10.65125	9.4374
H	−5.96108	12.28661	5.48517
H	−1.18474	1.39534	7.25273
C	−7.18291	13.58911	−0.52618
C	−6.70435	12.83621	8.05613
H	−8.14384	13.63707	−0.05794
H	−6.62726	14.4721	−0.28986
O	−7.35001	13.49422	−1.94548
H	−7.35211	13.22454	7.29822
H	−7.28508	12.56241	8.91409
O	−5.74794	13.83396	8.42245
H	−7.83198	14.26095	−2.26485
H	−6.20581	14.60977	8.75538

Figure S2 DOS diagram representation of **H1** (top) and **H1-Zn** (bottom). The singlet transitions with the highest oscillator strengths are shown on the spectra with black solid lines.

Figure S3 FT-IR spectrum of the compound chalcone.

Figure S4 ^1^H-NMR spectrum of **H1** in DMSO-d_6_ (500 MHz).

Figure S5 ^13^C NMR spectrum of **H1** in DMSO-d_6_ (125 MHz).

Figure S6 FTIR spectrum of **H1**.

Figure S7 HRMS-TOF-ESI spectrum of **H1**.

Figure S8 ^1^H NMR spectrum of **H1-Zn** in DMSO-d_6_ (500 MHz).

Figure S9 ^13^C NMR spectrum of **H1-Zn** in DMSO-d_6_ (125 MHz).

Figure S10 FTIR spectrum of **H1-Zn**.

Figure S11 HRMS-TOF-ESI spectrum of **H1-Zn**.

Figure S12 Optimized structure of **H1-Zn** (left) and molecular electrostatic potential (right).

Figure S13 Theoretical UV-VIS spectra of **H1** (top) and **H1-Zn** (bottom).

## Figures and Tables

**Scheme:**
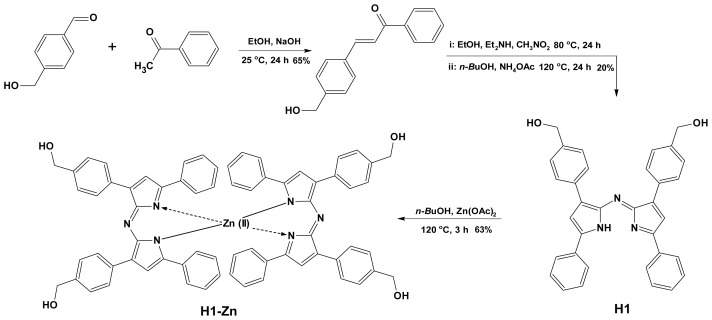
Synthesis of the target molecules: free-base azadipyrromethene (**H1**) and zinc(II) complex (**H1-Zn**).

**Table 1:** Photophysical parameters of the synthesized compounds in THF.

Compound	l_abs_ (max/nm)	e [M^−1^cm^−1^] x10^3^)	FWHM (nm)	f_F_ a	Photofading rate contants *k* (×10^−3^ mol/h) b	Φ_Δ_ c
**H** **_1_**	600	36.8	80	0.0054	2.14	0.127
**H** **_1_** **-Zn**	594/647	87.4/56.6	98	0.0023	1.39	0.233

a The emission spectra of the compounds contain significant scattering peaks. Peak intensities and hence fluorescence quantum yields are negligible.

b Rate constant for photostability.

c Methylene blue (Φ_Δ_ = 0.57 in CH_2_Cl_2_) was used as the reference compound for determining singlet oxygen quantum yields.

**Table 2:** Interfragment charge transfer analysis of compounds **H1** and **H1-Zn** for the electronic transition S_0_ → S_1_ in the gas phase.

Compounds	Transferred electrons between fragments			CT%	LE%
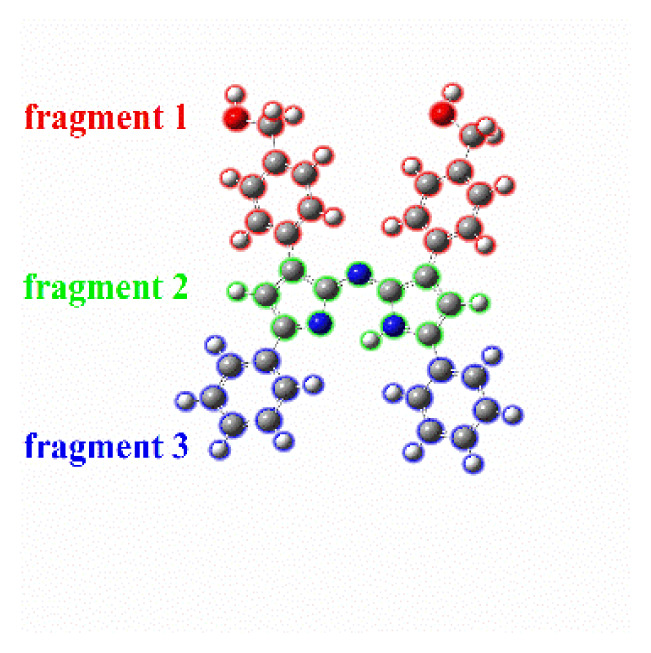	1 → 2: 0.2602 1 ← 2: 0.0478 *Net*: 0.2124 (1 → 2)	1 → 3: 0.0582 1 ← 3: 0.0113 *Net*: 0.0469 (1 → 3)	2 → 3: 0.0873 2 ← 3: 0.0926 *Net*: −0.00530 (2 → 3)	56	44
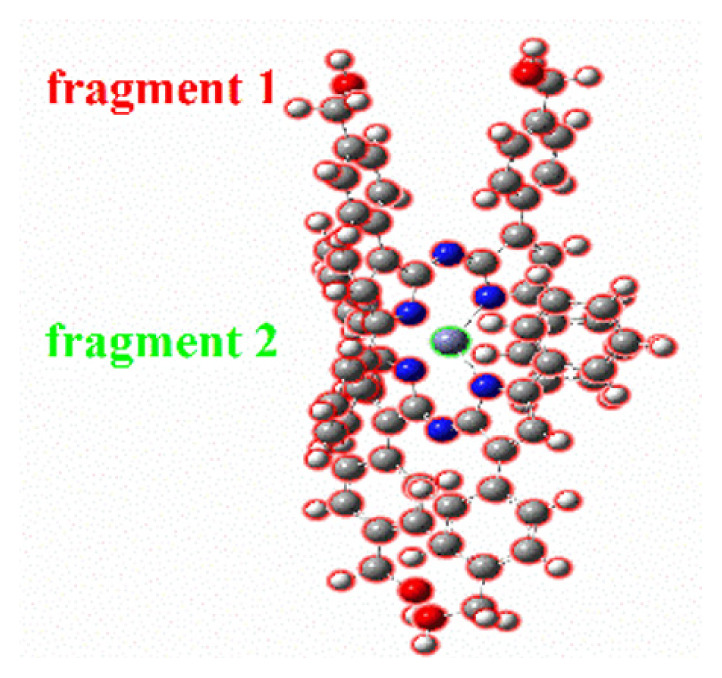	1 → 2: 0.00250 1 ← 2:0.00002 *Net*: 0.00247 (1 → 2)			99	1

**Table 3:** Decomposition temperatures and thermal stability of the compounds.

Compound	T _x_(°C)*	Decomposition rate (%/min)	**T10% (°C)	T30% (°C)	T50% (°C)
**H1**	307 674 1051	1.60 1.77 1.91	298	586	714
**H1-Zn**	297 514 891	1.39 2.28 1.12	277	436	528

* Maximum decomposition temperatures based on DTG plot.

** T10%: temperature at which 10% of initial mass is lost, and so on.
