# Supplementary figures and images for: Photophysical, thermal, and DFT studies on a tetraaryl-azadipyrromethene ligand and its zinc(II) complex
Source: Turk J Chem. 2023 Oct 10;47(6):1438–51. doi: 10.55730/1300-0527.3626 (PMC10965187; doi:10.55730/1300-0527.3626)

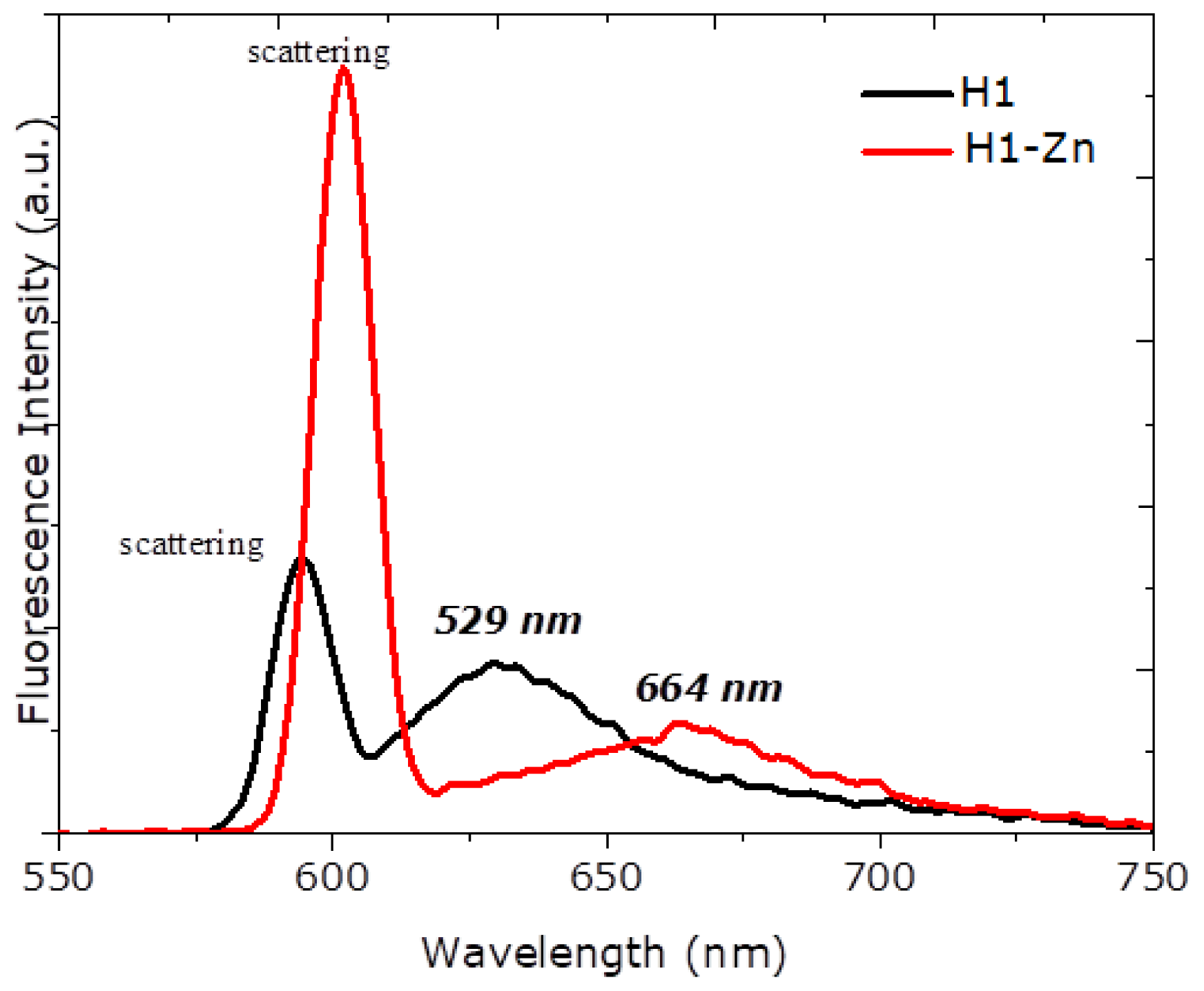

Supplement: Figure S1 — Fluorescence profiles of the compounds in THF. [file tjc-47-06-1438s1.tif]

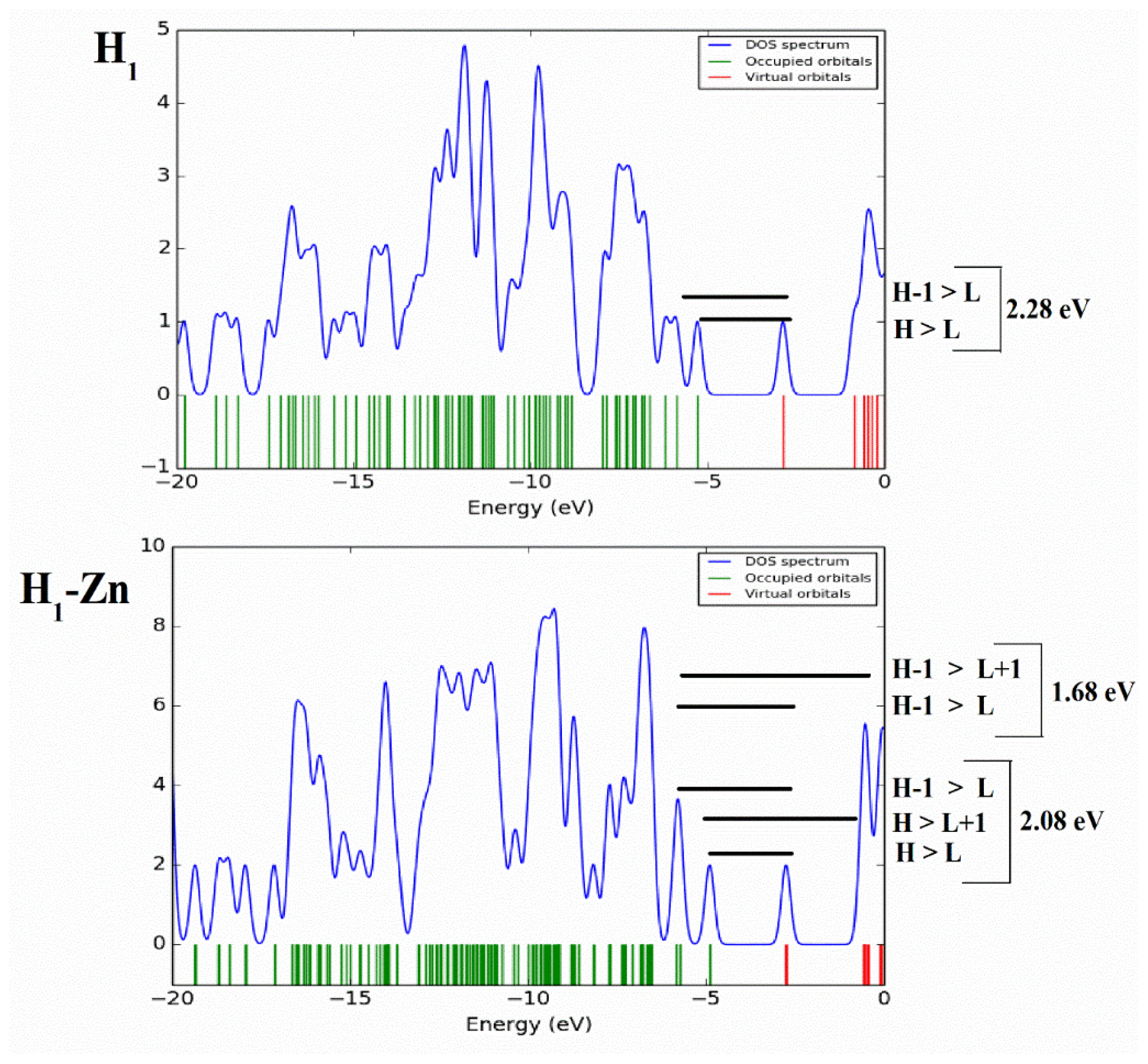

Supplement: Figure S2 — DOS diagram representation of H1 (top) and H1-Zn (bottom). The singlet transitions with the highest oscillator strengths are shown on the spectra with black solid lines. [file tjc-47-06-1438s2.tif]

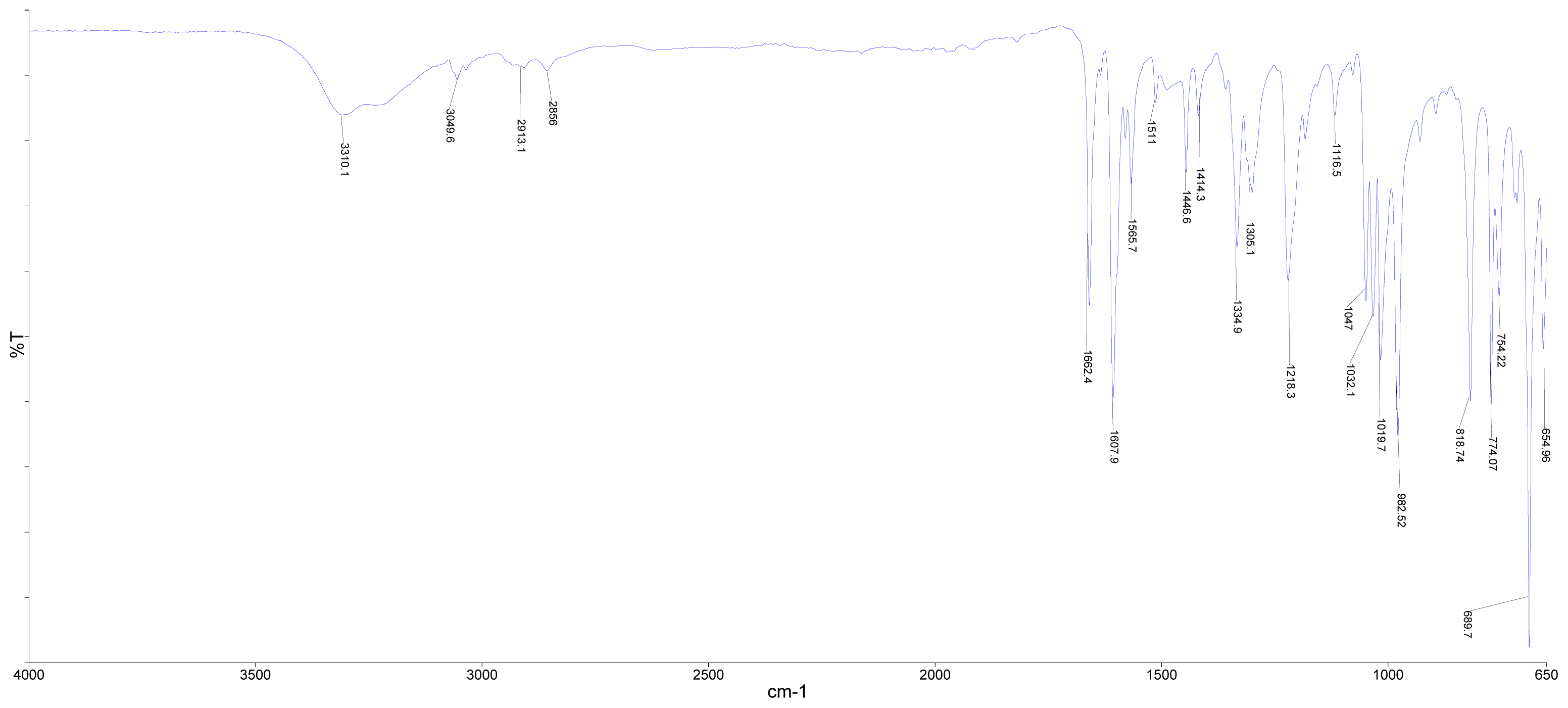

Supplement: Figure S3 — FT-IR spectrum of the compound chalcone. [file tjc-47-06-1438s3.tif]

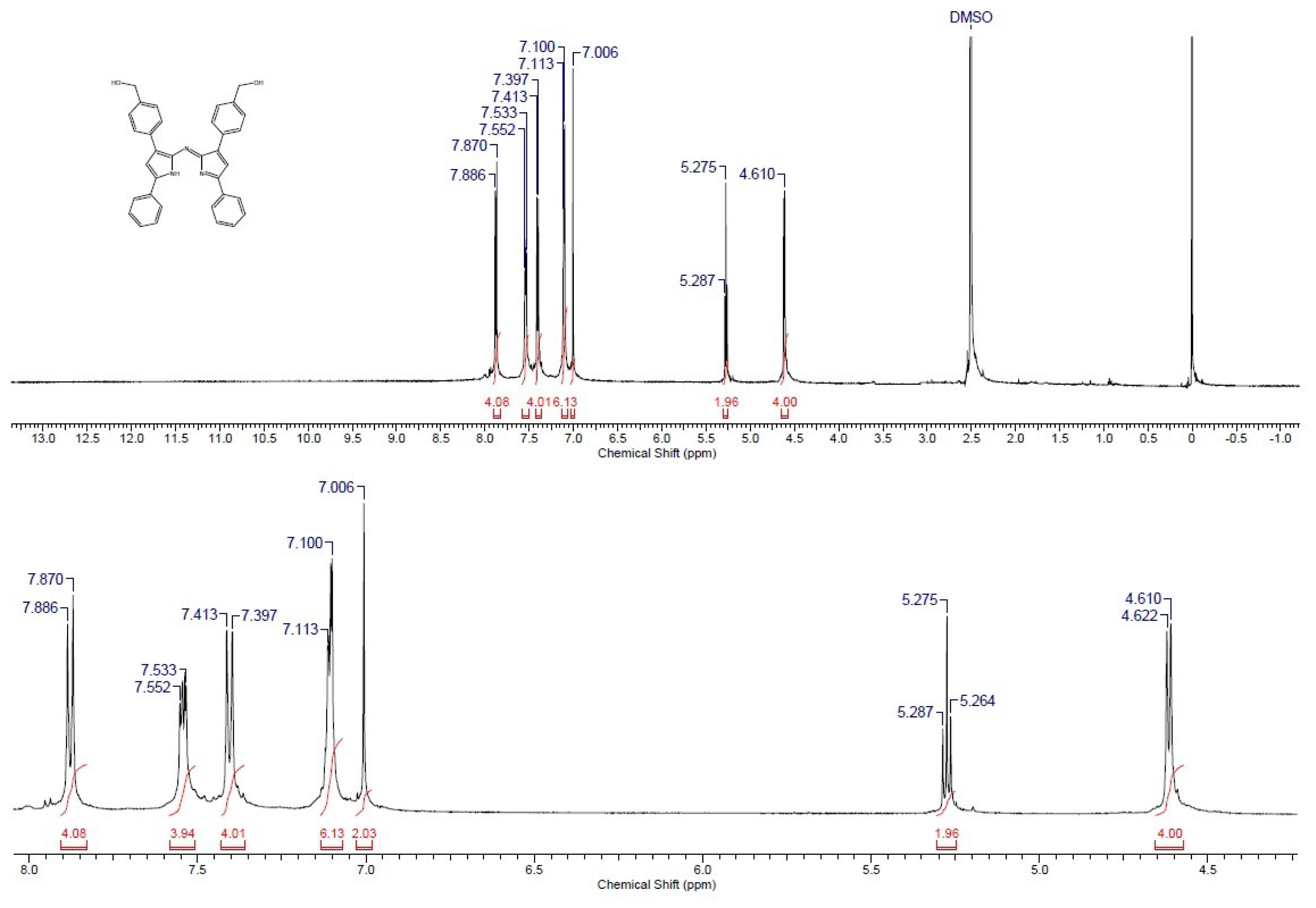

Supplement: Figure S4 — 1H-NMR spectrum of H1 in DMSO-d6 (500 MHz). [file tjc-47-06-1438s4.tif]

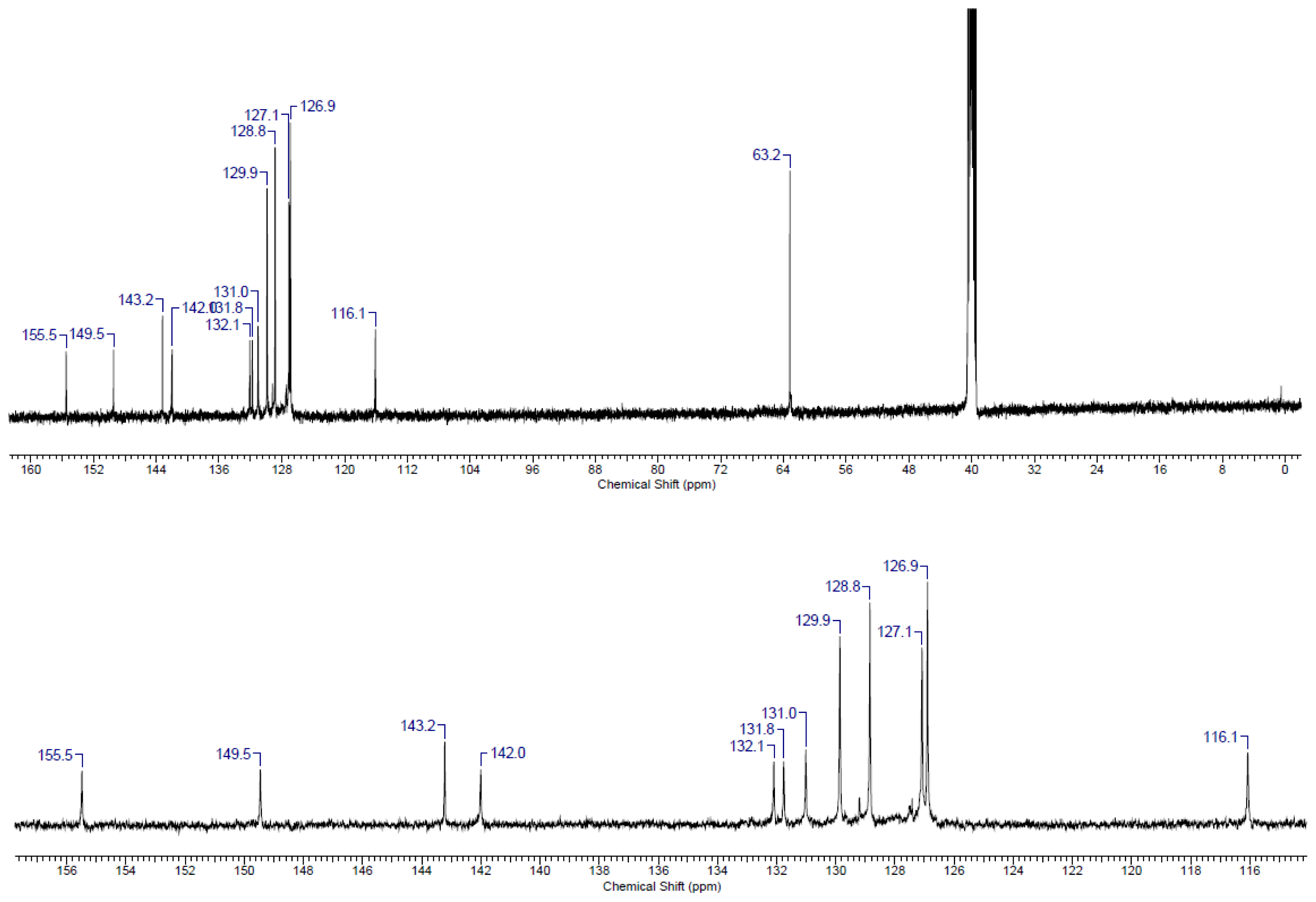

Supplement: Figure S5 — 13C NMR spectrum of H1 in DMSO-d6 (125 MHz). [file tjc-47-06-1438s5.tif]

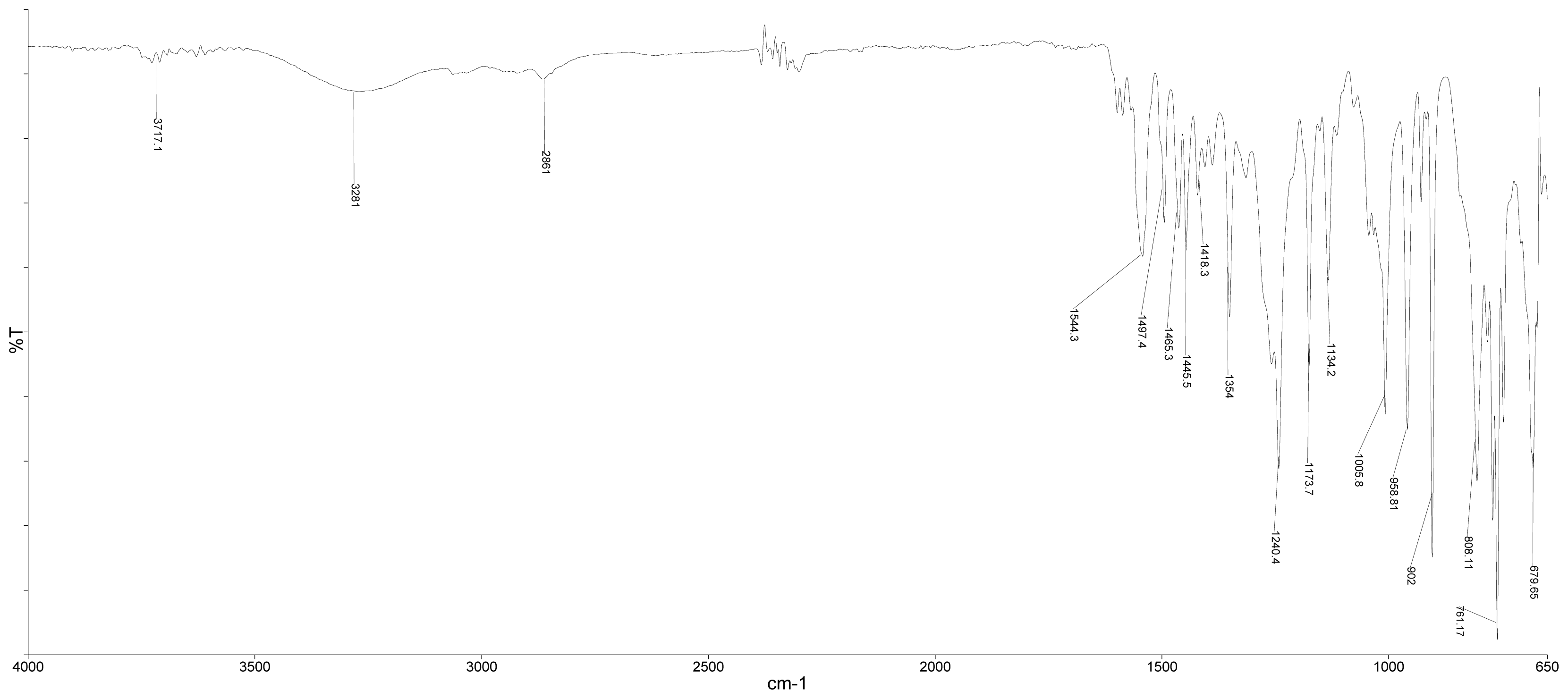

Supplement: Figure S6 — FTIR spectrum of H1. [file tjc-47-06-1438s6.tif]

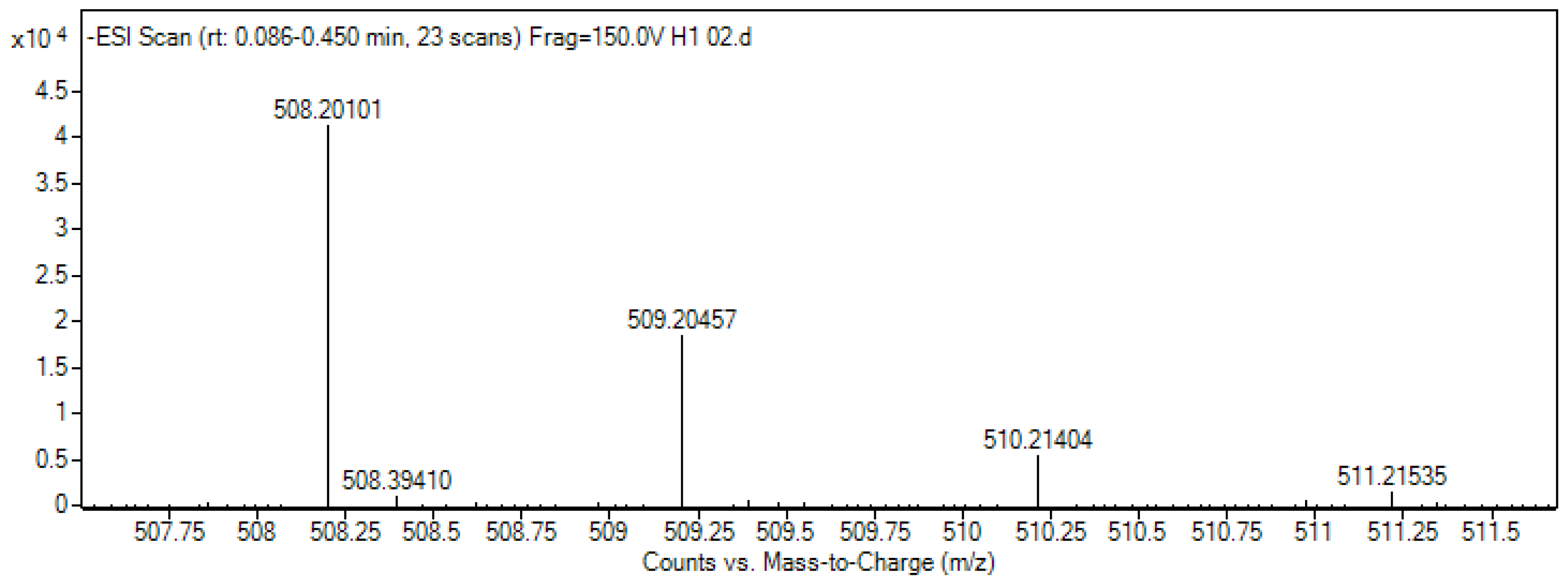

Supplement: Figure S7 — HRMS-TOF-ESI spectrum of H1. [file tjc-47-06-1438s7.tif]

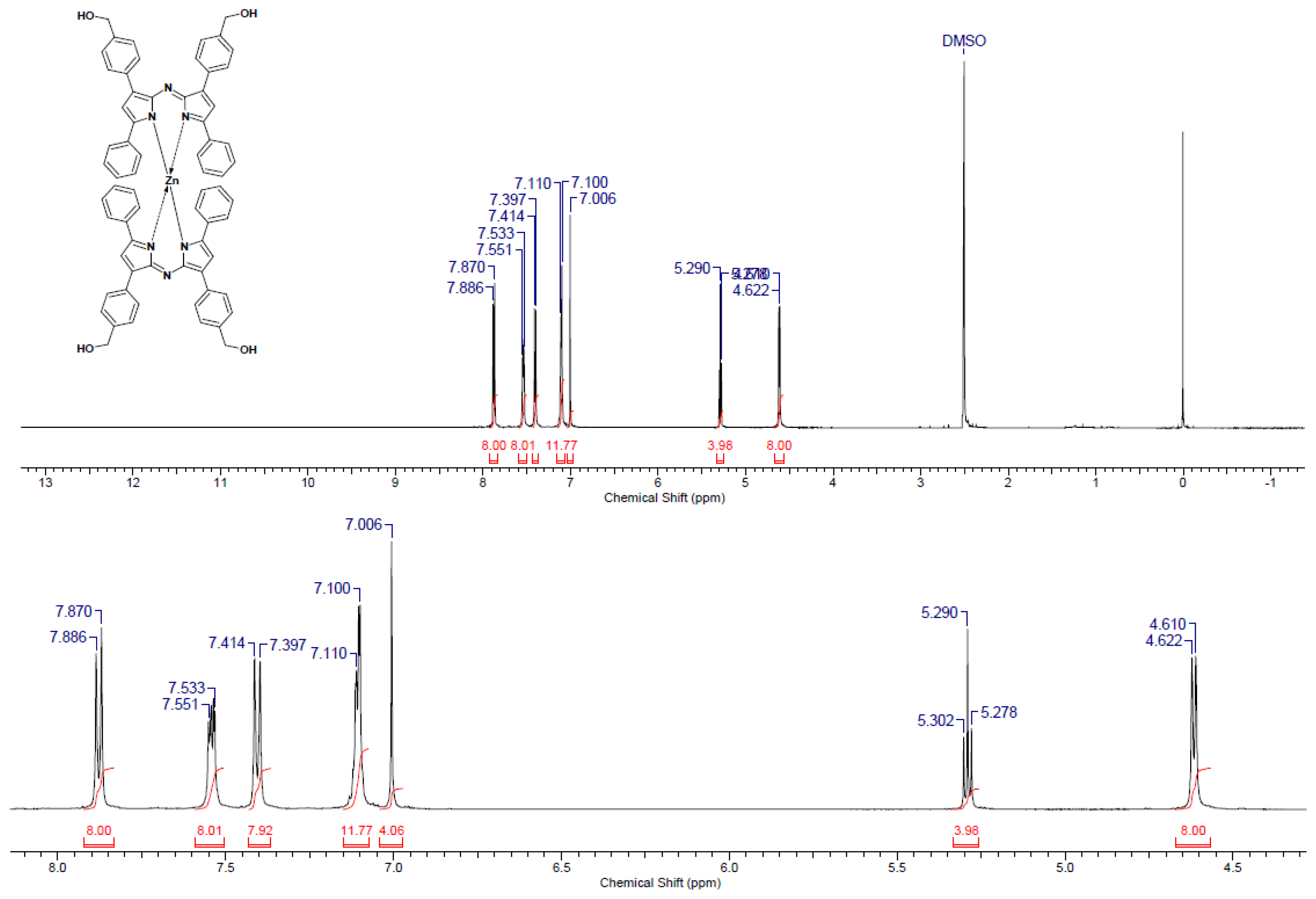

Supplement: Figure S8 — 1H NMR spectrum of H1-Zn in DMSO-d6 (500 MHz). [file tjc-47-06-1438s8.tif]

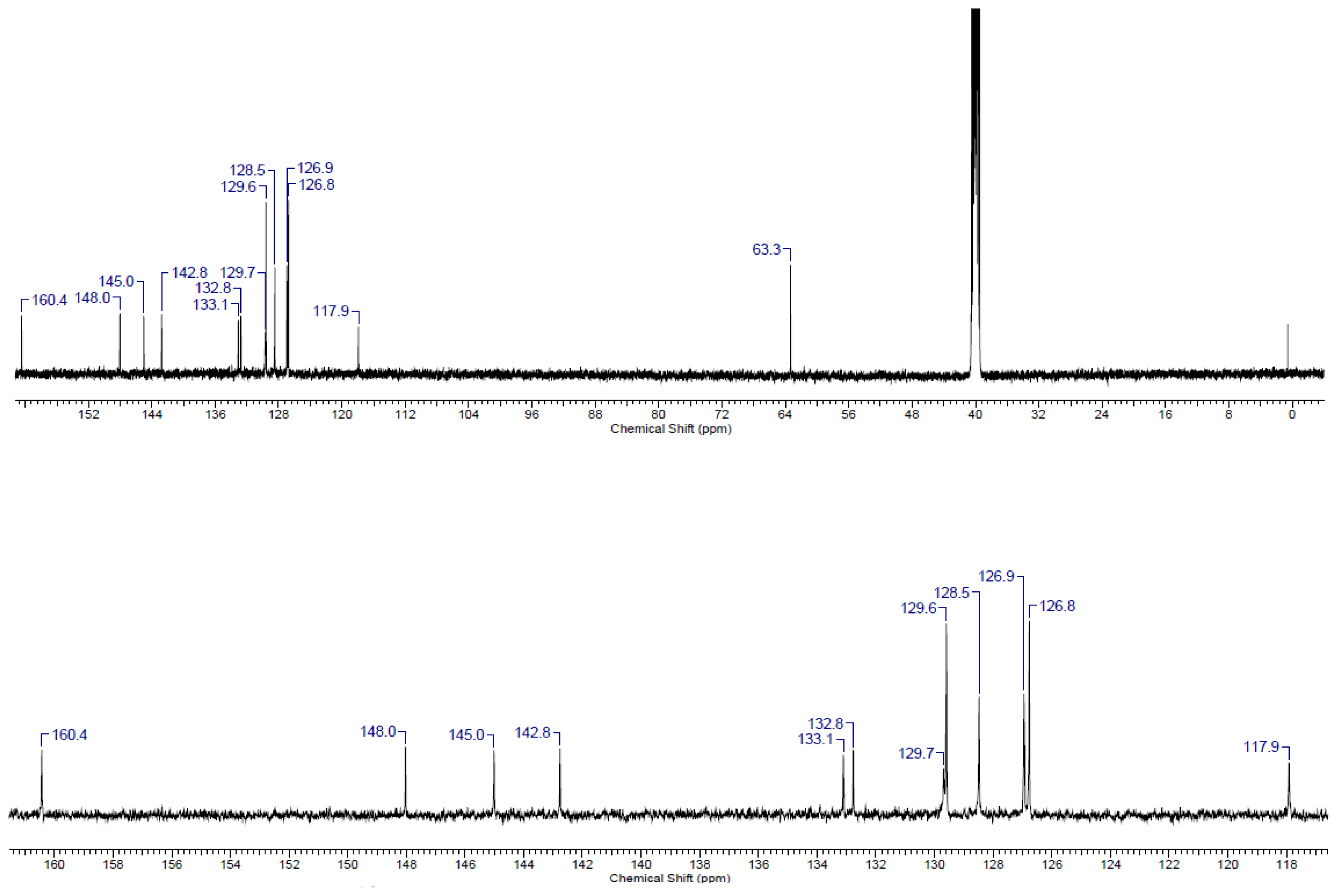

Supplement: Figure S9 — 13C NMR spectrum of H1-Zn in DMSO-d6 (125 MHz). [file tjc-47-06-1438s9.tif]

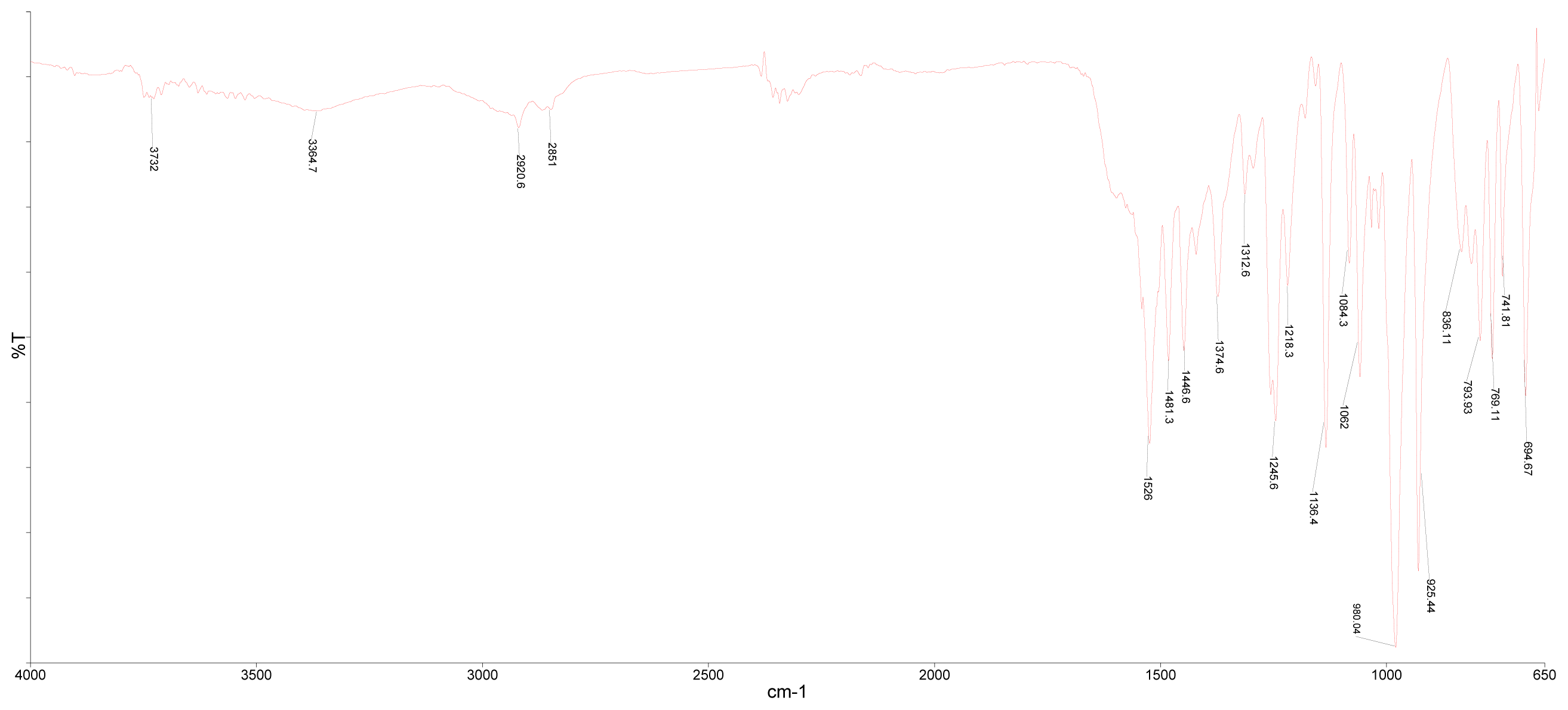

Supplement: Figure S10 — FTIR spectrum of H1-Zn. [file tjc-47-06-1438s10.tif]

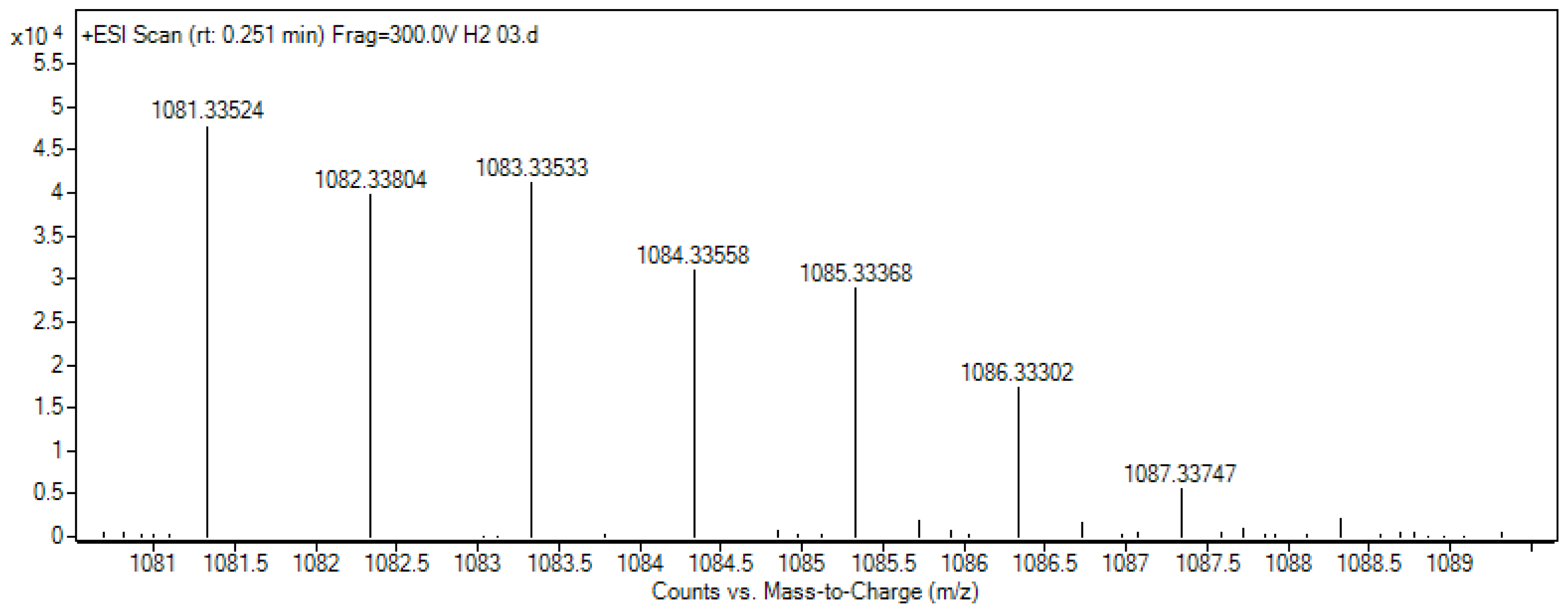

Supplement: Figure S11 — HRMS-TOF-ESI spectrum of H1-Zn. [file tjc-47-06-1438s11.tif]

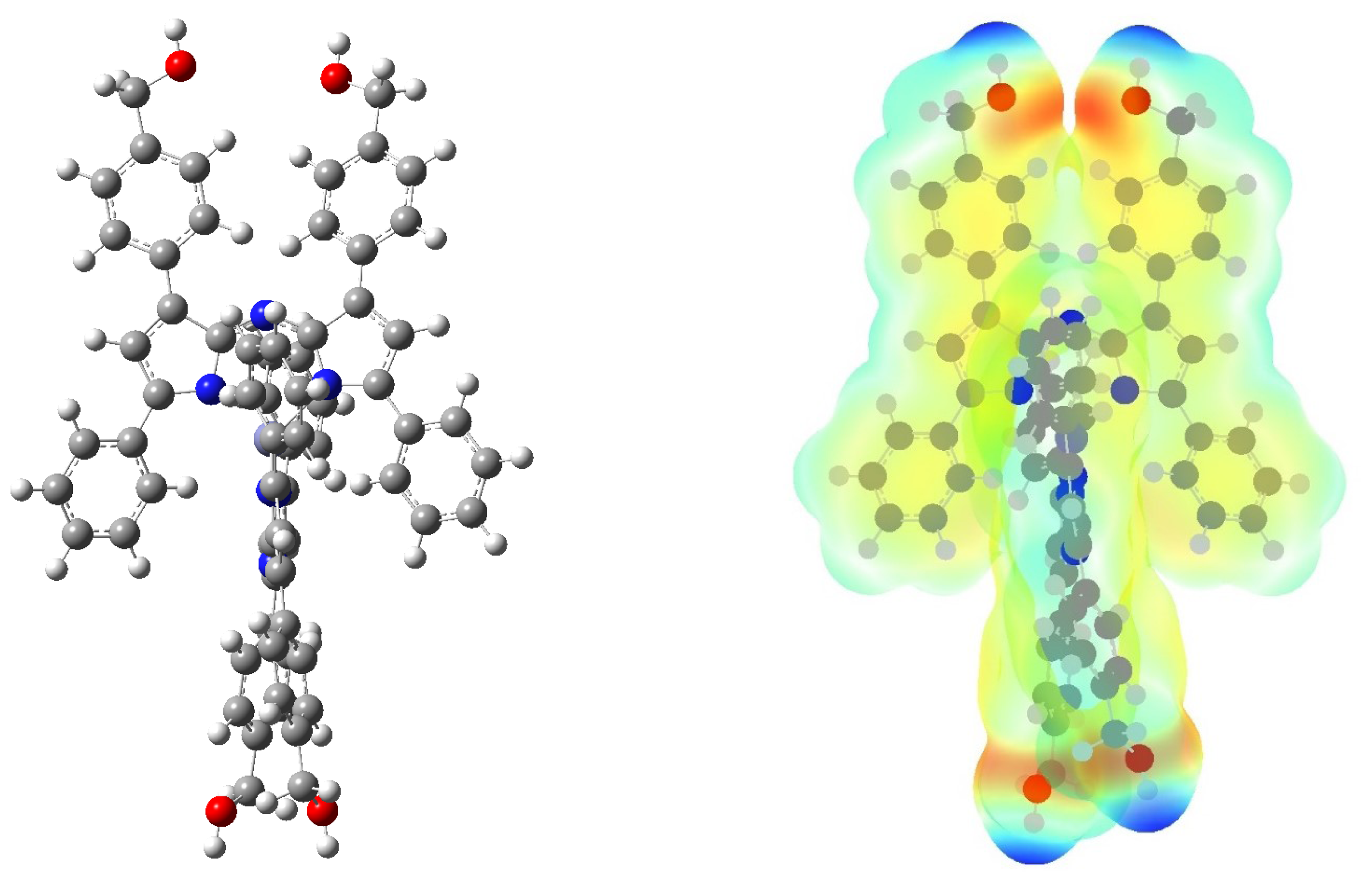

Supplement: Figure S12 — Optimized structure of H1-Zn (left) and molecular electrostatic potential (right). [file tjc-47-06-1438s12.tif]

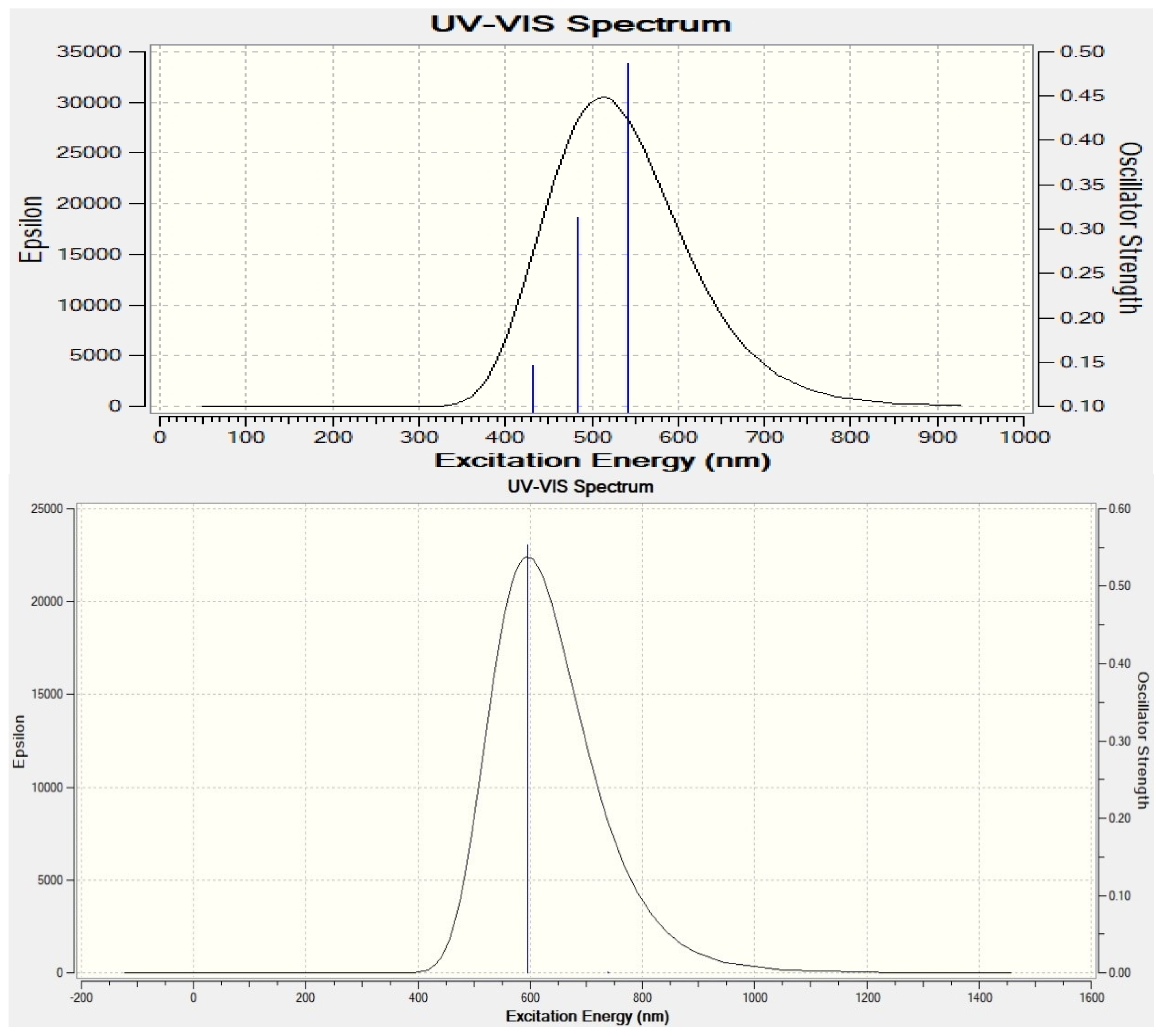

Supplement: Figure S13 — Theoretical UV-VIS spectra of H1 (top) and H1-Zn (bottom). [file tjc-47-06-1438s13.tif]
